# Phenolic profiles and in vitro biochemical properties of Thai herb ingredients for chronic diseases prevention

**DOI:** 10.1038/s41598-023-49074-5

**Published:** 2023-12-07

**Authors:** Nattira On-Nom, Sirinapa Thangsiri, Woorawee Inthachat, Piya Temviriyanukul, Piyapat Trisonthi, Chaowanee Chupeerach, Dalad Siriwan, Uthaiwan Suttisansanee

**Affiliations:** 1 Food and Nutrition Academic and Research Cluster, Institute of Nutrition, Mahidol University, Salaya, Phutthamonthon, Nakhon Pathom, 73170 Thailand; 2 Institute of Food Research and Product Development, Kasetsart University, Chatuchak, Bangkok, 10900 Thailand

**Keywords:** Biochemistry, Chemical biology, Metabolic disorders

## Abstract

Traditional remedies using natural ingredients have been handed down over generations, providing collective information for the development of modern therapeutics. These natural products have a long history of safe consumption with curative effects but lack of scientific-based evidence hinders the mass production of new remedies containing active ingredients with particular medicinal properties. This research investigated the phenolic profiles and biochemical properties of 29 herbal ingredients identified in two traditional Thai remedies, Prasachandaeng (PSCD) and Chantaharuethai (CHRT), and their effectiveness in combating chronic diseases. These two traditional remedies are used to reduce fever but some ingredients have been previously reported to possess other health-related properties. Statistical analysis by TOPSIS indicated that *Biancaea sappan* (L.) Tod. extract exhibited the highest overall closeness coefficient (C) score analyzed from all variables including total phenolic contents, antioxidant potentials, and enzyme inhibitions. *Bouea macrophylla* Griff. extract showed potential as an effective agent against diabetes through inhibition of two carbohydrate degrading enzymes, α-glucosidase and α-amylase, while *Dischidia major* (Vahl) Merr. showed strong properties as an anti-angiotensin-converting enzyme, leading to the control of hypertension. *Dracaena cochinchinensis* (Lour.) S.C. Chen effectively controlled the progression of Alzheimer’s disease through the inhibition of cholinergic and β-amyloid formation enzymes. These results can be used as preliminary data for the development of new remedies to prevent or treat particular chronic diseases.

## Introduction

Traditional remedies are developed using collective knowledge gained from a long history of trial and error to achieve products with high efficacy and low adverse effects. Folk medicines are an important resource for modern drug and food supplement development, such as artemisinin from *Artemisia annua* L. as an anti-malaria drug^1^, *Gingko biloba* L. extract as a brain boosting supplement according to Chinese medical scriptures^2^, Triphala as a rejuvenating agent according to Ayurvedic medicine^3^, and aspirin from *Salix alba* bark extract according to Sumerian folk medicine^4^. Thai traditional remedies including Prasachandaeng (PSCD) and Chantaharuethai (CHRT) are listed in Taxila pharmacopeia, comprising knowledge on types of fever and proper treatments, and have been used as alternative treatments for COVID-19 fever and other non-communicable diseases (NCDs).

PSCD and CHRT were formulated according to The National List of Essential Drugs: List of herbal medicine products (Food and Drug Administration of Thailand)^5^. PSCD comprises 12 ingredients including *Dracaena cochinchinensis* (Lour.) S.C. Chen, *Myristica fragrans* Houtt., *Heliciopsis terminalis* (Kurz) Sleumer, *Ligusticum sinense* Oliv., *Kaempferia galanga* L., *Biancaea sappan* (L.) Tod., *Citrus* x *aurantifolia* (Christm.) Swingle, *Bouea macrophylla* Griff., *Nelumbo nucifera* Gaertn., *Mesua ferrea* L., *Jasminum sambac* (L.) Aiton, and *Mammea siamensis* (Miq.) T. Anderson. Traditionally, these herbs are used as antipyretics, herbal tonics and detoxification agents. This formula was proven for the efficacy to inhibit inflammatory mediators in RAW.264 cells and suppress LPS induced fever in animal model without evidence of liver damage^6^. CHRT is also an antipyretic, especially for fever with skin rash and seizure, consisting of 27 ingredients including *Dracaena cochinchinensis* (Lour.) S.C. Chen, *Tarenna hoaensis* Pit., *Myristica fragrans* Houtt., *Euphorbia antiquorum* L., *Mimusops elengi* L., *Urceola minutiflora* (Pierre) D.J. Middleton, *Carissa spinarum* L., *Gynura pseudochina* (L.) DC., *Glycyrrhiza glabra* L., *Tacca chantrieri* André, *Calamus longisetus* Griff., *Angelica dahurica* (Hoffm.) Benth. & Hook.f. ex Franch. & Sav., *Artemisia annua* L., *Dischidia major* (Vahl) Merr., *Picrorhiza kurroa* Royle ex Benth., *Ligusticum sinense* Oliv., *Mesua ferrea* L., *Mimusops elengi* L., *Mammea siamensis* (Miq.) T. Anderson, *Jasminum sambac* (L.) Aiton, *Nelumbo nucifera* Gaertn., *Dendrobium crumenatum* Sw., *Sophora exigua* Craib, *Enhalus acoroides* (L.f.) Royle, *Aristolochia pierrei* Lecomte, *Hibiscus surattensis* L. (musk), and *Pogostemon cablin* (Borneo camphor). However, scientific-based data about this remedy formula are limited, with scant previous reports on health efficacy against NCDs of individual ingredients in PSCD and CHRT. For example, *D. cochinchinensis* wood extract inhibited formation of amyloid-β and induced neuronal cell regeneration^7^. Flavonoid derivatives isolated from the stem resin of this plant were also reported for anti-thrombin activity^8^. *Myristica fragrans* seed extract showed a downregulated effect on the expression of lipid genes, resulting in anti-obesity activity and preventing non-alcoholic fatty liver disease^9^, while a root extract of *A. dahurica* showed vasorelaxant activity and attenuated hypertension in animal model^10^. *Nelumbo nucifera* pollen extract inhibited aldose reductase, the enzyme associated with diabetic complication^11^, while many parts of this plant exhibited inhibitory effect against many key enzymes relevant to Alzheimer’s disease, hypertension, obesity, and diabetes^12,13^.

This study investigated the phenolic compositions, antioxidant activities, and in-hibitory potential of each ingredient in PSCD and CHRT remedies against key enzymes relevant to NCDs including Alzheimer’s disease (β-secretase (BACE-1), butyrylcholin-esterase (BChE) and acetylcholinesterase (AChE)), diabetes mellitus (α-glucosidase and α-amylase), and hypertension (angiotensin-converting enzyme (ACE)). Results provide valuable data for further development of new remedies for the prevention and treatment of NCDs through key enzyme inhibitions, thereby supporting the conservation of traditional healthcare wisdom.

## Materials and methods

### Sample selection, preparation, and extraction

The study samples were purchased as dried herbs from Charoensuk Pharma Supply Co., Ltd. (Phrapathomchedi district, Nakhon Pathom, Thailand) in October 2021. The sample collection was conducted following the guidelines and regulations of the legislation of Thailand. Identification and authentication were kindly assisted by Dr. Prachaya Srisanga (Taxonomist and Herbarium Curator, The Botanical Garden Organization, Chiang Mai, Thailand) and Assoc. Prof. Dr. Chusie Trisonthi (Taxonomist, Faculty of Science, Chiang Mai University, Chiang Mai, Thailand). The combination of ingredients in each remedy is shown in Supplementary Table 1, with physical appearance in Supplementary Table 2. Three ingredients in CHRT including *Aristolochia pierrei* Lecomte, *Hibiscus surattensis* L. (musk), and *Pogostemon cablin* (Borneo camphor) were not investigated because the first was previously reported for its toxicity^14^, while the latter two were added only for their fragrance. Plant parts of the dry samples were deposited at the Sireeruckhachati Nature Learning Park, Mahidol University (Nakhon Pathom, Thailand) with voucher specimens as shown in Table 1.

**Table 1:** Scientific names, abbreviations, plant parts and voucher specimen of twenty-nine herbal ingredients used in this research.

Scientific names	Abbreviation	Plant parts	Voucher specimen
*Jasminum sambac* (L.) Aiton	JS	Flower	PBM-006049
*Mammea siamensis* (Miq.) T. Anderson	MS	Flower	PBM-006051
*Mesua ferrea* L	MF	Flower	PBM-006048
*Mimusops elengi* L	MEF	Flower	PBM-006065
*Nelumbo nucifera* Gaertn	NN	Stamen	PBM-006047
*Calamus longisetus* Griff	CL	Stem	PBM-006060
*Dendrobium crumenatum* Sw	DC	Stem	PBM-006066
*Urceola minutiflora* (Pierre) D.J. Middleton	UN	Stem	PBM-006055
*Tacca chantrieri* André	TC	Whole plant	PBM-006059
*Biancaea sappan* (L.) Tod	CSA	Heartwood	PBM-006043
*Carissa spinarum* L	CSP	Heartwood	PBM-006056
*Dracaena cochinchinensis* (Lour.) S.C. Chen	DL	Heartwood	PBM-006039
*Euphorbia antiquorum* L	EAN	Heartwood	PBM-006053
*Mimusops elengi* L	MET	Heartwood*	PBM-006054
*Myristica fragrans* Houtt	MF	Heartwood	PBM-006041
*Tarenna hoaensis* Pit	TH	Heartwood	PBM-006052
*Enhalus acoroides* (L.f.) Royle	EAC	Rhizome	PBM-006050
*Gynura pseudochina* (L.) DC	GP	Rhizome	PBM-006057
*Kaempferia galanga* L	KG	Rhizome	PBM-006042
*Ligusticum sinense* Oliv	LS	Rhizome	PBM-006040
*Picrorhiza kurroa* Royle ex Benth	PK	Rhizome	PBM-006064
*Angelica dahurica* (Hoffm.) Benth. & Hook.f. ex Franch. & Sav	AD	Root	PBM-006061
*Artemisia annua* L	AA	Root	PBM-006062
*Bouea macrophylla* Griff	BM	Root	PBM-006045
*Citrus x aurantifolia* (Christm.) Swingle	CA	Root	PBM-006044
*Dischidia major* (Vahl) Merr	DM	Root	PBM-006063
*Glycyrrhiza glabra* L	GA	Root	PBM-006058
*Heliciopsis terminalis* (Kurz) Sleumer	HT	Root	PBM-006046
*Sophora exigua* Craib	SE	Root	PBM-006067

*Rotten wood.

The herbs were ground into a fine powder (50 mesh) and kept at − 20 °C until extraction. Powdery samples (1 g) were extracted using 70% (*v/v*) ethanol (50 mL) and macerated for 72 h. The filtrate was obtained using Whatman No.3 filter paper (Whatman International Ltd., Maidstone, UK) through a Büchner funnel. The filtrate was dried using a rotary vacuum evaporator (Rotavapor® R-215, BÜCHI Labortechnik, Flawil, Switzerland), with the water bath temperature set to 40 °C. All dried extracts were stored at − 20 °C and protected from light for further experiments.

### Analysis of phenolic profile

Phenolic profiles of all herbs were investigated by liquid chromatography-electrospray ionization tandem mass spectrometry (LC–ESI–MS/MS), while a well-established protocol along with LC–ESI–MS/MS parameters and validations was previously reported (with data in Supplementary Table 3)^15,16^. The dried extracts were redissolved in 62.5% (*v/v*) methanol and filtered through a 0.22 µm polytetrafluoroethylene (PTFE) syringe filter. The filtrate was loaded into a LC–ESI–MS/MS system consisting of a 2.1 mm × 100 mm, 2.6 μm Accucore RP-MS column, a Dionex Ultimate 3000 series ultrahigh-performance liquid chromatograph (UHPLC), a TSQ Quantis Triple Quadrupole mass spectrometer (MS), a diode array detector and a Chromeleon 7 chromatography data system (Thermo Fisher Scientific, Bremen, Germany). The gradient mobile phase used in this experiment is shown in Table 2.

**Table 2:** A gradient mobile phase used for a liquid chromatography-electrospray ionization tandem mass spectrometry (LC–ESI–MS/MS) analysis.

Time (min)	Solvent A	Solvent B	Flow rate
0.0–8.0	10–80%	90–20%	0.5 mL/min
8.0–8.1	80–10%	20–90%	
8.1–10.0	10%	90%	

Solvent A: acetonitrile; solvent B: Milli-Q water (18.2 MΩ·cm resistivity at 25 °C) containing 0.1% (*v/v*) formic acid.

Twenty-four phenolics were used as the standards including quercetin (> 98.0% HPLC, E), hesperidin (> 90.0% HPLC, T), apigenin (> 98.0% HPLC), genistein (> 98.0% HPLC), 3,4-dihydroxybenzoic acid (≥ 97% T), ( −)-epigallocatechin gallate (> 98.0% HPLC), kaempferol (> 97.0% HPLC), 4-hydroxybenzoic acid (> 99.0% GC, T), *p*-coumaric acid (> 98.0% GC, T), naringenin (> 93.0% HPLC, T), chlorogenic acid (> 98.0% HPLC, T), ferulic acid (> 98.0% GC, T), syringic acid (> 97.0% T), cinnamic acid (> 98.0% HPLC), luteolin (> 98.0% HPLC), myricetin (> 97.0% HPLC), sinapic acid (> 99.0% GC, T) and caffeic acid (> 98.0% HPLC, T) from Tokyo Chemical Industry (Tokyo, Japan), isorhamnetin (≥ 99.0% HPLC) from Extrasynthese (Genay, France), gallic acid (97.5–102.5% T), rutin (≥ 94% HPLC), and galangin (≥ 98.0% HPLC) from Wuhan ChemFaces Biochemical Co., Ltd. (Hubei, China), vanillic acid (≥ 97% HPLC), and rosmarinic acid (≥ 98% HPLC) from Sigma-Aldrich (St. Louis, MO, USA). The chromatograms of the phenolic standards and the herbal extracts are presented in Supplementary Fig. 1.

Total phenolic contents (TPCs) of the extract were also determined utilizing a well-established protocol as previously reported without any modification^17^. Folin-Ciocalteu’s phenol was used as a reagent and gallic acid concentration ranging 0–200 µg/mL was used as a standard. Absorbance at 765 nm was detected utilizing a Synergy™ HT 96-well UV–visible microplate reader and Gen 5 data analysis software (BioTek Instruments, Inc., Winooski, VT, USA). A calibration curve of gallic acid was used to generate a linear Eq. (1) with coefficient of determination (R^2^) of 0.9979,

1 $$y = \, 0.006x + \, 0.0377$$

where *y* is the absorbance at 765 nm, and *x* is the concentration of the gallic acid standard. Results were expressed as mg gallic acid equivalent (GAE)/g dry weight (DW).

### Analysis of antioxidant activities

Antioxidant activities of the herbal extracts were determined using three assays including 2,2-diphenyl-1-picrylhydrazyl (DPPH) radical scavenging, ferric ion reducing antioxidant power (FRAP), and oxygen radical absorbance capacity (ORAC) assays as previously reported without any modification^17^. All chemicals and reagents were purchased from Sigma-Aldrich (St. Louis, MO, USA). Assay types, main reagents, and detection wavelengths are shown in Table 3. The reaction was detected using a microplate reader, and Trolox was used as a standard, with results expressed as µmol Trolox equivalent (TE)/g DW.

**Table 3:** The components of 2,2-diphenyl-1-picrylhydrazyl (DPPH) radical scavenging, ferric ion reducing antioxidant power (FRAP) and oxygen radical absorbance capacity (ORAC) assays.

Antioxidant assay	Type of assay	Reagents	Detection wavelength
DPPH radical scavenging assay	End-point	DPPH radical solution	520 nm
FRAP assay	End-point	FRAP reagent containing FeCl_3_·6H_2_O solution, 2,4,6-tri(2-pyridyl)-s-triazine and acetate buffer	600 nm
ORAC assay	Kinetics	2,2’-Azobis(2-amidinopropane) dihydrochloride and sodium fluorescein	λ_ex_ = 485 nm, λ_em_ = 528 nm

### Analysis of enzyme inhibitory activities

The enzyme inhibitory activities of the herbal extracts were performed using AChE, BChE, BACE-1, ACE, α-amylase, and α-glucosidase assays using well-established protocols^18–21^, as summarized in Table 4. The AChE, BChE, α-amylase, and α-glucosidase inhibitory assays were kinetically measured using a Synergy™ HT UV–visible microplate reader and Gen 5 data analysis software (BioTek Instruments, Inc., Winooski, VT, USA), while the same instruments were used to determine the end-point inhibitory assays of BACE-1 and ACE. All chemicals and reagents were purchased from Sigma-Aldrich (St. Louis, MO, USA), with percentage of inhibition calculated using Eq. (2) as follows:

2 $$\% {\text{ inhibition }} = \left( {1 - \frac{B - b}{{A - a}}} \right) \times 100,$$

where *A* is the initial velocity (*V*_*0*_) of a reaction with an enzyme but without a herbal extract (control), *a* is the *V*_*0*_ of the reaction without an enzyme and a herbal extract (control blank), *B* is the *V*_*0*_ of a reaction with an enzyme and a herbal extract (sample), and *b* is the *V*_*0*_ of a reaction with a herbal extract but without an enzyme (sample blank).

**Table 4:** The assay components including an enzyme, a substrate, an indicator, a herbal extract and a detection wavelength for enzyme inhibitory assays.

Types of enzyme assay	Content of main ingredients in enzyme assay					
	Enzyme content	Substrate content	Indicator	Extract		Detection wavelength
AChE	100 μL of 0.25 µg/mL AChE ^1^	50 μL of 0.32 mM ACh	10 µL of 16 mM DTNB	40 µL		412 nm
BChE	100 μL of 1.5 µg/mL BChE ^2^	50 μL of 0.4 mM BCh				
BACE-1	Following manufacturer's recommendations of BACE-1 FRET assay kit (Sigma-Aldrich, St. Louis, MO, USA)					λ_ex_ = 320 nm λ_em_ = 405 nm
ACE	9 µL of 0.5 U/mL ACE ^3^	90 µL of 3 mM HHL	45 µL of 20 mg/mL PDA	150 µL		λ_ex_ = 360 nm λ_em_ = 485 nm
α-Amylase	100 µL of 50 mg/mL α-amylase ^4^	50 µL of 30 mM pNPM		50 µL		405 nm
α-Glucosidase	10 µL of 0.2 U/mLα-glucosidase ^5^	25 µL of 10 mMpNPG + 160 µL KPB (pH 7)		5 µL		

^1^*Electrophorus electricus* acetylcholinesterase (AChE, 1000 units/mg); ^2^equine serum butyrylcholinesterase (BChE, ≥ 10 units/mg); ^3^rabbit lung angiotensin-converting enzyme (ACE, ≥ 2 units/mg protein); ^4^porcine pancreatic α-amylase (type VII, ≥ 10 unit/mg); ^5^*Saccharomyces cerevisiae* α-glucosidase (type I, ≥ 10 U/mg protein). DTNB: 5,5′-dithiobis(2-nitrobenzoic acid); ACh: acetylthiocholine; BCh: butyrylthiocholine; BACE-1: β-secretase; FRET: fluorescence resonance energy transfer; HHL:* N*-hippuryl-His-Leu tetrahydrate; PDA:* O*-phthaldialdehyde; pNPM: *p*-nitrophenyl-α-D-maltohexaoside; KPB: potassium phosphate buffer; pNPG: *p*-nitrophenyl-α-D-glucopyranoside.

### Statistical analysis

All experiments were performed in triplicate for three independent sets of samples (*n* = 3). Statistical analysis was determined utilizing one-way analysis of variance (ANOVA) with Duncan's multiple range test and significant differences set at *p* < 0.05. Principal component analysis (PCA) and hierarchical cluster analysis (HCA) of TPCs, antioxidant potentials, and key enzyme inhibitory activities were determined using XLSTAT® (Addinsoft Inc., New York, NY, USA). The Technique for Order Preference by Similarity to Ideal Solution (TOPSIS) comprehensive evaluation analysis was analyzed according to previous reports^22^.

## Results

### Phenolic profiles and total phenolic contents

Phenolic profiles of all the herbal extracts were investigated utilizing LC–ESI–MS/MS with 24 authentic standards. Eight phenolic acids were detected in all extracts, as indicated in Table 5. Among the detected phenolic acids, most herbal extracts contained 3,4-dihydroxybenzoic acid (16 herbal extracts), followed by caffeic acid (10 herbal extracts), gallic acid and chlorogenic acid (8 herbal extracts), *p*-coumaric acid (4 herbal extracts), 4-hydroxybenzoic acid (2 herbal extracts), and rosmarinic acid and ferulic acid (1 herbal extract). Results indicated that MF exhibited the highest amounts of gallic acid and 3,4-dihydroxybenzoic acid, while chlorogenic acid was abundantly found in CSP. Highest content of 4-hydroxybenzoic acid was detected in NN, caffeic acid in PK, and *p*-coumaric in DC. Rosmarinic acid was only found in AA, while ferulic acid was only detected in LS. Overall, MF exhibited the highest phenolic acid contents using this analytical method, while no phenolic acids were detected in MET, KG, CA, GA, HT, and SE.

**Table 5:** Phenolic acid profile of twenty-nine herbal extracts detected by a liquid chromatography-electrospray ionization tandem mass spectrometry (LC–ESI–MS/MS).

Abbreviation	Phenolic acids (µg/g extract)							
	Gallic acid	3,4-Dihydroxybenzoic acid	Chlorogenic acid	4-Hydroxybenzoic acid	Caffeic acid	Rosmarinic acid	Ferulic acid	*p*-Coumaric acid
JS	ND	ND	ND	670.66 ± 8.52^bA^	369.12 ± 1.28^fC^	ND	ND	515.45 ± 10.12^bB^
MS	930.93 ± 17.80^cdeB^	7121.65 ± 0.00^bA^	ND	ND	215.24 ± 2.54^iC^	ND	ND	223.99 ± 1.02^dC^
MF	ND	277.55 ± 2.51^i^	ND	ND	ND	ND	ND	ND
MEF	1161.21 ± 43.52^cA^	448.19 ± 9.37^gB^	ND	ND	220.35 ± 1.17^iC^	ND	ND	240.65 ± 3.68^cC^
NN	798.47 ± 11.48^deC^	1091.54 ± 11.43 dB	ND	2239.53 ± 19.78^aA^	ND	ND	ND	ND
CL	ND	335.77 ± 4.61 h	ND	ND	ND	ND	ND	ND
DC	685.88 ± 40.61^eC^	1703.24 ± 11.37^cA^	ND	ND	ND	ND	ND	995.93 ± 3.56^aB^
UN	ND	236.62 ± 1.40^ijB^	368.35 ± 11.83^eA^	ND	ND	ND	ND	ND
TC	ND	552.79 ± 26.01^f^	ND	ND	ND	ND	ND	ND
CSA	ND	161.73 ± 1.31^kB^	429.32 ± 5.15^dA^	ND	ND	ND	ND	ND
CSP	ND	218.37 ± 14.14^jC^	1893.96 ± 42.98^aA^	ND	626.11 ± 9.41 dB	ND	ND	ND
DL	ND	ND	ND	ND	ND	ND	ND	ND
EAN	246.13 ± 9.35^fA^	221.56 ± 1.70^jB^	ND	ND	ND	ND	ND	ND
MET	ND	ND	ND	ND	ND	ND	ND	ND
MF	12,341.14 ± 395.84^aA^	10,027.51 ± 107.66^aB^	ND	ND	ND	ND	ND	ND
TH	ND	122.70 ± 0.20^kC^	852.07 ± 8.36^cA^	ND	333.23 ± 8.16^gB^	ND	ND	ND
EAC	ND	845.52 ± 10.66^e^	ND	ND	ND	ND	ND	ND
GP	ND	ND	135.40 ± 1.92^gB^	ND	956.87 ± 10.43^cA^	ND	ND	ND
KG	ND	ND	ND	ND	ND	ND	ND	ND
LS	ND	ND	271.46 ± 1.03^fC^	ND	612.81 ± 1.62 dB	ND	3532.09 ± 111.35^A^	ND
PK	ND	ND	ND	ND	3286.25 ± 4.26^a^	ND	ND	ND
AD	ND	ND	90.26 ± 0.31^hB^	ND	313.75 ± 0.90^hA^	ND	ND	ND
AA	ND	ND	1460.20 ± 25.73^bA^	ND	1318.03 ± 10.29^bB^	481.63 ± 1.71^C^	ND	ND
BM	2148.36 ± 5.32^bA^	1057.82 ± 15.71^dB^	ND	ND	ND	ND	ND	ND
CA	ND	ND	ND	ND	ND	ND	ND	ND
DM	963.75 ± 3.03^cdA^	151.94 ± 1.43^kB^	ND	ND	ND	ND	ND	ND
GA	ND	ND	ND	ND	ND	ND	ND	ND
HT	ND	ND	ND	ND	ND	ND	ND	ND
SE	ND	ND	ND	ND	ND	ND	ND	ND

All data are denoted as mean ± standard deviation (SD) of triplicate experiments (*n* = 3). List of abbreviation of samples are shown in Table 1. Different lowercase letters indicate significantly different contents of the same phenolic detected in different samples and different capital letters indicate significantly different contents of different phenolics detected in the same sample at *p* < 0.05 using one-way analysis of variance (ANOVA) and Duncan’s multiple comparison test. ND, not detected.

Seven flavonoids were detected in the herbal extracts (Table 6). Most herbal extracts contained naringenin (17 herbal extracts), followed by rutin (9 herbal extracts), luteolin and apigenin (7 herbal extracts), isorhamnetin (6 herbal extracts), kaempferol (2 herbal extracts)**,** and hesperidin (1 herbal extract). Naringenin was abundantly found in TC, while rutin was high in JS. PK contained the highest luteolin content, and apigenin was highly detected in AA. NN exhibited the highest contents of isorhamnetin and kaempferol, while hesperidin was only detected in CA. Overall, CA exhibited the highest flavonoid contents using this analytical method, while no phenolic acids were detected in CSP, MET, TH, GP, KG, LS, AD, and HT.

**Table 6:** Flavonoid profile of twenty-nine herbal extracts detected by a liquid chromatography-electrospray ionization tandem mass spectrometry (LC–ESI–MS/MS).

Abbreviation	Flavonoids (µg/g extract)						
	Rutin	Luteolin	Apigenin	Naringenin	Kaempferol	Isorhamnetin	Hesperidin
JS	389.88 ± 2.21^aA^	ND	ND	9.59 ± 0.92^iB^	ND	7.96 ± 0.65^eB^	ND
MS	8.28 ± 0.52^dE^	452.30 ± 14.29^cB^	729.70 ± 8.42^bA^	247.37 ± 7.08^eC^	43.41 ± 3.23^bD^	ND	ND
MF	ND	69.24 ± 0.75^e^	ND	ND	ND	ND	ND
MEF	ND	ND	ND	65.66 ± 3.63^gA^	ND	8.90 ± 0.62^eB^	ND
NN	49.89 ± 2.52^cC^	ND	ND	63.53 ± 5.15^gC^	2439.38 ± 8.44^aA^	1412.61 ± 12.82^aB^	ND
CL	ND	ND	ND	588.67 ± 41.19^c^	ND	ND	ND
DC	75.75 ± 3.49^bC^	ND	ND	684.52 ± 9.29^bA^	ND	221.63 ± 2.37^bB^	ND
UN	3.86 ± 0.14^e^	ND	ND	ND	ND	ND	ND
TC	ND	75.12 ± 1.87^eB^	7.23 ± 0.60^fC^	1992.84 ± 13.41^aA^	ND	ND	ND
CSA	ND	ND	ND	9.58 ± 0.28^i^	ND	ND	ND
CSP	ND	ND	ND	ND	ND	ND	ND
DL	ND	ND	610.66 ± 59.68^cA^	41.18 ± 2.97^hB^	ND	ND	ND
EAN	ND	ND	ND	325.15 ± 2.76^d^	ND	ND	ND
MET	ND	ND	ND	ND	ND	ND	ND
MF	77.03 ± 2.84^bD^	265.12 ± 4.37^dA^	126.22 ± 2.81^dB^	9.38 ± 0.15^iE^	ND	19.77 ± 1.54^dC^	ND
TH	ND	ND	ND	ND	ND	ND	ND
EAC	3.85 ± 0.01^eA^	ND	ND	3.12 ± 0.40^iB^	ND	ND	ND
GP	ND	ND	ND	ND	ND	ND	ND
KG	ND	ND	ND	ND	ND	ND	ND
LS	ND	ND	ND	ND	ND	ND	ND
PK	ND	2269.97 ± 64.24^aA^	138.90 ± 6.06^dB^	ND	ND	ND	ND
AD	ND	ND	ND	ND	ND	ND	ND
AA	ND	1686.81 ± 35.69^bB^	7113.28 ± 35.13^aA^	95.46 ± 7.76^fD^	ND	166.45 ± 2.52^cC^	ND
BM	ND	109.79 ± 1.78^eA^	ND	55.23 ± 4.30^ghB^	ND	ND	ND
CA	6.69 ± 0.15^deB^	ND	ND	ND	ND	ND	20,370.94 ± 331.25 ^A^
DM	5.37 ± 0.39^deB^	ND	ND	16.71 ± 0.40^iA^	ND	ND	ND
GA	ND	ND	69.32 ± 3.89^eB^	317.22 ± 6.67^dA^	ND	ND	ND
HT	ND	ND	ND	ND	ND	ND	ND
SE	ND	ND	ND	39.58 ± 0.63^h^	ND	ND	ND

All data are denoted as mean ± standard deviation (SD) of triplicate experiments (*n* = 3). List of abbreviation of samples are shown in Table 1. Different lowercase letters indicate significantly different contents of the same phenolic detected in different samples and different capital letters indicate significantly different contents of different phenolics detected in the same sample at *p* < 0.05 using one-way analysis of variance (ANOVA) and Duncan’s multiple comparison test. ND, not detected.

When combining phenolic acids and flavonoid contents, MF exhibited the highest phenolic content detected by the LC–ESI–MS/MS method, while no phenolics were detected in MET, KG, and HT. In comparison to TPCs analyzed by spectrophotometric methods using Folin-Ciocalteu’s phenol as the reagent, TPCs of all herbal extracts ranged 26.31–728.50 mg GAE/g extract with CSA exhibiting the highest TPC, while MET gave the lowest (Table 7).

**Table 7:** Total phenolic contents (TPCs) and antioxidant activities determined by 2,2-diphenyl-1-picrylhydrazyl (DPPH) radical scavenging, ferric ion reducing antioxidant power (FRAP) and oxygen radical absorbance capacity (ORAC) assays of twenty-nine herbal extracts.

Abbreviation	TPCs (mg GAE/g extract)	Antioxidant Activities (µmol TE/g extract)		
		DPPH radical scavenging assay	FRAP assay	ORAC assay
JS	77.33 ± 1.21^kl^	0.33 ± 0.01^jk^	267.50 ± 7.32^jk^	1513.56 ± 13.26^ij^
MS	123.05 ± 1.59^hi^	0.54 ± 0.02^n^	630.29 ± 5.79^g^	1222.44 ± 78.23^jkl^
MF	150.72 ± 2.29^hi^	0.74 ± 0.02^e^	532.65 ± 11.12^h^	2251.19 ± 102.19^g^
MEF	147.64 ± 5.20^hi^	0.90 ± 0.07^d^	959.28 ± 11.52^f^	1252.22 ± 114.89^jkl^
NN	146.53 ± 0.31^hi^	0.69 ± 0.02^ef^	1019.24 ± 12.41^f^	1059.11 ± 45.46^klm^
CL	66.14 ± 0.55^lm^	0.39 ± 0.02^ij^	294.55 ± 7.70^j^	940.54 ± 8.67 l^mn^
DC	76.86 ± 1.35^kl^	0.32 ± 0.02^jk^	321.52 ± 20.78^j^	1871.06 ± 186.49^h^
UN	416.94 ± 27.89^c^	1.99 ± 0.16^b^	2742.61 ± 38.53^d^	5282.87 ± 350.47^b^
TC	190.75 ± 6.25^f^	0.58 ± 0.02^fgh^	748.94 ± 14.38^g^	3206.79 ± 213.41^f^
CSA	728.50 ± 12.83^a^	1.80 ± 0.14^c^	3836.52 ± 104.45^b^	15,906.87 ± 728.09^a^
CSP	179.83 ± 1.16^g^	0.65 ± 0.02^efg^	973.33 ± 43.71^f^	3119.16 ± 149.55^f^
DL	140.94 ± 4.49^i^	0.57 ± 0.01^gh^	543.33 ± 15.89^h^	3635.56 ± 239.76^e^
EAN	69.19 ± 1.25^lm^	0.49 ± 0.00^hi^	405.45 ± 7.99^i^	1040.81 ± 83.45^klm^
MET	26.31 ± 0.68^q^	0.16 ± 0.00^no^	127.68 ± 7.07^m^	244.27 ± 14.74^p^
MF	123.14 ± 2.96^j^	0.71 ± 0.06^e^	957.00 ± 20.37^f^	2951.25 ± 40.77^f^
TH	175.25 ± 1.28^g^	0.51 ± 0.01^h^	996.36 ± 37.63^f^	4130.67 ± 284.21^d^
EAC	290.39 ± 4.22^e^	0.85 ± 0.08^d^	1391.97 ± 13.89^e^	4843.94 ± 132.92^c^
GP	41.53 ± 1.18^p^	0.12 ± 0.00^o^	135.07 ± 5.15^m^	327.22 ± 23.34^op^
KG	47.73 ± 0.58^nop^	0.14 ± 0.01^o^	190.22 ± 15.86^klm^	336.05 ± 6.04^op^
LS	44.31 ± 2.23^op^	0.19 ± 0.02^mno^	128.77 ± 2.63^m^	614.05 ± 29.43^no^
PK	82.97 ± 1.64^k^	0.31 ± 0.00^jkl^	308.71 ± 8.47^j^	1745.53 ± 28.63^hi^
AD	54.89 ± 0.09^no^	0.15 ± 0.00^no^	156.23 ± 4.04^m^	1052.00 ± 89.03^klm^
AA	66.63 ± 2.73^lm^	0.26 ± 0.01^klmn^	276.38 ± 3.34^j^	1084.67 ± 33.21^klm^
BM	485.28 ± 3.28^b^	1.89 ± 0.12^c^	3192.17 ± 87.59^c^	4036.92 ± 233.91^d^
CA	51.98 ± 0.91^nop^	0.19 ± 0.01^mno^	327.46 ± 3.37^j^	1377.79 ± 151.06^jk^
DM	355.28 ± 3.06^d^	2.35 ± 0.14^a^	5196.96 ± 70.45^a^	2242.46 ± 182.50^g^
GA	113.28 ± 1.57^j^	0.30 ± 0.00^jklm^	177.27 ± 11.25^lm^	1974.40 ± 105.11^gh^
HT	58.61 ± 1.23^mn^	0.20 ± 0.01^lmno^	314.13 ± 12.80^j^	731.15 ± 40.44^mn^
SE	152.78 ± 9.35^h^	0.34 ± 0.01^jk^	253.64 ± 3.37^jkl^	858.50 ± 57.26^mn^

All data are denoted as mean ± standard deviation (SD) of triplicate experiments (*n* = 3). List of abbreviation of samples are shown in Table 1. Different superscript letters indicate significantly different antioxidant activities determined by the same assay of different herbs (*p* < 0.05) using one-way analysis of variance (ANOVA) and Duncan’s multiple comparison test. GAE: gallic acid equivalent; TE: Trolox equivalent.

### Antioxidant activities

The antioxidant activities of all herbal extracts were investigated utilizing DPPH radical scavenging, FRAP, and ORAC assays. The first two assays follow single electron transfer (SET) mechanism of antioxidants, while the last follows the hydrogen atom transfer (HAT) mechanism. Results in Table 7 (with raw data in Supplementary Table 4) indicated that all herbal extracts exhibited different degrees of antioxidant activities, ranging 0.12–2.35 µmol TE/g extract for the DPPH radical scavenging assay, with DM exhibiting the highest activities and the lowest detected in GP and KG. All herbal extracts also exhibited FRAP activities ranging 156.23–5196.96 µmol TE/g extract, with DM exhibiting the highest FRAP activity and MET, GP, and AD the lowest FRAP activity providers. The ORAC activities of all herbal extracts ranged 244.27–15,906.87 µmol TE/g extract. Similar to TPCs, CSA exhibited the highest ORAC activity, while MET gave the lowest.

### Enzyme inhibitory activities

The inhibitory activities of the herbal extracts were assessed on the key enzymes that control the occurrence of some NCDs including type II diabetes (T2DM) (α-amylase and α-glucosidase), Alzheimer’s disease (AChE, BChE, and BACE-1) and hypertension (ACE). Results indicated that the herbal extracts exhibited different degrees of inhibition against these enzymes using particular extract concentrations, as indicated in Table 8.

**Table 8:** Inhibitory activities against α-amylase, α-glucosidase, acetylcholinesterase (AChE), butyrylcholinesterase (BChE), β-secretase (BACE-1) and angiotensin-converting enzyme (ACE) of 29 herbal extracts.

Abbreviation	Enzyme inhibitory activities (% inhibition)					
	α-Amylase^1^	α-Glucosidase^2^	AChE^3^	BChE^3^	BACE-1^3^	ACE^4^
JS	ND	4.79 ± 0.22^m^	46.29 ± 1.28^jk^	52.46 ± 0.49^e^	32.03 ± 2.19^m^	ND
MS	ND	83.84 ± 1.41^f^	95.91 ± 3.18^ab^	95.80 ± 1.03^a^	28.25 ± 2.49^n^	70.85 ± 0.15^d^
MF	76.06 ± 1.81^f^	93.05 ± 0.79^d^	94.73 ± 1.44^ab^	96.91 ± 0.39^a^	41.29 ± 1.58^ij^	74.98 ± 0.20^c^
MEF	84.23 ± 0.69^cd^	87.48 ± 5.57^e^	98.55 ± 0.36^a^	87.48 ± 5.57^b^	16.77 ± 1.21^p^	79.06 ± 0.55^b^
NN	87.16 ± 0.10^bc^	98.52 ± 0.03^a^	99.01 ± 0.03^a^	95.39 ± 0.70^a^	48.16 ± 1.12^g^	65.21 ± 0.61^f^
CL	45.69 ± 1.84^j^	93.51 ± 0.28^cd^	78.65 ± 0.70^d^	65.25 ± 1.49^d^	36.25 ± 3.54^kl^	60.50 ± 0.99^h^
DC	ND	28.82 ± 0.75^k^	59.07 ± 1.13^h^	38.19 ± 1.54^g^	21.73 ± 2.16^o^	21.32 ± 1.01m
UN	57.27 ± 2.27^h^	94.83 ± 0.12^bcd^	98.72 ± 0.24^a^	97.68 ± 0.34^a^	20.83 ± 0.86^o^	79.46 ± 0.91^b^
TC	39.51 ± 3.59^k^	73.60 ± 1.20^g^	74.17 ± 1.99^e^	42.88 ± 1.32^f^	31.88 ± 2.99^m^	76.05 ± 0.23^c^
CSA	79.36 ± 3.09^e^	96.67 ± 1.28^abc^	95.32 ± 0.47^ab^	96.67 ± 1.28^a^	7.80 ± 0.28^q^	36.99 ± 0.29^l^
CSP	12.90 ± 1.41^m^	38.93 ± 1.30^j^	58.80 ± 4.25^h^	35.80 ± 3.29^g^	29.94 ± 2.71^mn^	ND
DL	85.97 ± 0.90^c^	96.26 ± 0.15^abcd^	91.60 ± 7.63^bc^	96.26 ± 0.15^a^	66.87 ± 1.35^c^	57.60 ± 0.81^i^
EAN	ND	52.20 ± 1.41^i^	51.87 ± 4.19^j^	52.20 ± 1.41^e^	35.53 ± 2.27^l^	16.91 ± 0.51^o^
MET	ND	29.45 ± 1.69^k^	45.92 ± 2.49^kl^	ND	46.64 ± 2.48^gh^	46.01 ± 1.74^k^
MF	0.85 ± 0.09^o^	57.97 ± 1.46^h^	63.79 ± 1.46^g^	76.41 ± 0.79^c^	19.39 ± 0.64^op^	57.27 ± 0.35^i^
TH	ND	ND	52.55 ± 3.57^i^	ND	20.88 ± 1.66^o^	ND
EAC	92.60 ± 0.50^a^	95.20 ± 3.25^abcd^	98.70 ± 0.23^a^	91.22 ± 3.94^b^	ND	63.21 ± 0.73^g^
GP	85.54 ± 0.86^c^	4.63 ± 0.19^m^	41.15 ± 2.55^l^	ND	28.35 ± 2.37^n^	19.95 ± 0.80^n^
KG	ND	39.02 ± 1.79^j^	50.36 ± 3.12^ij^	23.23 ± 2.05^i^	61.47 ± 0.82^d^	ND
LS	89.95 ± 2.01^ab^	14.17 ± 1.46^l^	48.63 ± 1.44^ijk^	28.52 ± 1.73^h^	57.45 ± 0.41^e^	14.51 ± 0.87^p^
PK	53.80 ± 2.05^i^	ND	61.41 ± 6.10^gh^	97.27 ± 0.73^a^	35.76 ± 1.96^l^	54.24 ± 1.18^j^
AD	16.00 ± 1.40^m^	11.60 ± 0.68^l^	34.59 ± 2.32^m^	ND	43.96 ± 2.12^hi^	ND
AA	6.27 ± 0.46^n^	29.50 ± 1.33^k^	68.39 ± 0.37^f^	43.27 ± 2.23^f^	54.17 ± 1.24^f^	2.91 ± 0.25^q^
BM	92.66 ± 0.48^a^	97.45 ± 0.38^ab^	97.65 ± 0.07^a^	96.96 ± 1.42^a^	29.62 ± 1.20^mn^	80.86 ± 0.32^a^
CA	ND	27.74 ± 1.95^k^	52.39 ± 1.68^i^	24.47 ± 0.52^i^	75.53 ± 1.39^a^	17.01 ± 0.57^o^
DM	81.67 ± 0.47^de^	97.02 ± 0.19^ab^	90.12 ± 0.39^c^	98.57 ± 0.11^a^	60.19 ± 0.42^de^	81.19 ± 0.17^a^
GA	47.10 ± 2.90^j^	75.54 ± 2.48^g^	61.25 ± 0.38^gh^	75.26 ± 0.64^c^	71.60 ± 1.14^b^	67.51 ± 0.45^e^
HT	20.18 ± 1.94^l^	ND	64.30 ± 0.90^fg^	74.14 ± 0.61^c^	39.27 ± 2.20^jk^	ND
SE	67.16 ± 3.89^g^	87.84 ± 0.70^e^	96.74 ± 0.37^a^	88.85 ± 7.99^b^	65.12 ± 3.04^c^	67.18 ± 0.26^e^

All data are denoted as mean ± standard deviation (SD) of triplicate experiments (*n* = 3). List of abbreviation of samples are shown in Table 1. Different superscript letters indicate significantly different inhibitory activities of the same enzyme assay in different herbs (*p* < 0.05) using one-way analysis of variance (ANOVA) and Duncan’s multiple comparison test; AChE: acetylcholinesterase; BChE: butyrylcholinesterase; BACE-1: β-secretase; ACE: angiotensin-converting enzyme; ND: not detected; ^1^final extract concentration = 0.25 mg/mL; ^2^final extract concentration = 0.06 mg/mL; ^3^final extract concentration = 0.20 mg/mL; ^4^final extract concentration = 1 mg/mL.

Carbohydrate digestive enzymes, α-amylase and α-glucosidase, are the therapeutic targets for diabetic drug design, and acarbose is a competitive inhibitor of these enzymes. Twenty-one herbal extracts exhibited α-amylase inhibitory activities ranging 0.85–92.66% using extract concentration of 0.25 mg/mL, while inhibitory activities were not detected in 8 herbal extracts using the same extract concentration. Among the herbal extracts with α-amylase inhibitory activities, EAC and BM exhibited more than 90% inhibition, suggesting their high potentials as α-amylase inhibitors. These herbal extracts also exhibited higher degrees of inhibition against another carbohydrate degrading enzyme, α-glucosidase (ranging 4.63–98.52% inhibition using extract concentration of 0.06 mg/mL). Among the herbal extracts with α-glucosidase inhibitory activities, MF, NN, CL, UN, CSA, DL, EAC, BM, and DM exhibited more than 90% inhibition. Combining the data from these two carbohydrates hydrolyzing enzyme inhibitions, EAC and BM showed promise as antidiabetic agents.

Two hypotheses of the mechanisms of Alzheimer’s disease occurrence involve (i) termination of neurotransmitters by two cholinergic enzymes, AChE and BChE, and (ii) β-amyloid formation by BACE-1. Results indicated that at extract concentration of 0.20 mg/mL, all herbal extracts exhibited AChE inhibitory activities ranging 34.59–99.01%. Among these, MS, MF, MEF, NN, UN, CSA, DL, EAC, BM, DM, and SE exhibited AChE inhibitory activities at more than 90%. Likewise, their BChE inhibitory activities ranged 23.23–98.57% using extract concentration of 0.20 mg/mL. Among these, MS, MF, NN, UN, CSA, DL, EAC, PK, BM, and DM showed more than 90% inhibition, while no activity was detected in MET, TH, GP, and AD. Using the same extract concentration, BACE-1 inhibitory activities of 7.80–75.53% were observed in all herbal extracts, with the exception of EAC that had no inhibitory activity. CA exhibited the highest BACE-1 inhibitory activity but its AChE and BChE inhibitions were lower than 53%. When combining the inhibitory data from these three main enzymes that control Alzheimer’s disease occurrence, DL and DM had more than 90% inhibition against AChE and BChE and more than 60% inhibition against BACE-1 as two potential herbal extracts for reducing the risk of Alzheimer’s disease.

Inhibition of ACE, the main enzyme in reducing the risk of hypertension, was investigated using extract concentration of 1 mg/mL. Results indicated that all herbal extracts exhibited ACE inhibitory activities ranging 2.91–81.19% inhibition, with the exception of JS, CSP, TH, KG, AD, and HT with no ACE inhibitory activity detected. Among all the herbal extracts with detected ACE inhibitions, BM and DM exhibited more than 80% inhibition, suggesting their high potential as anti-ACE agents.

### Principal component analysis

**Figure 1:**
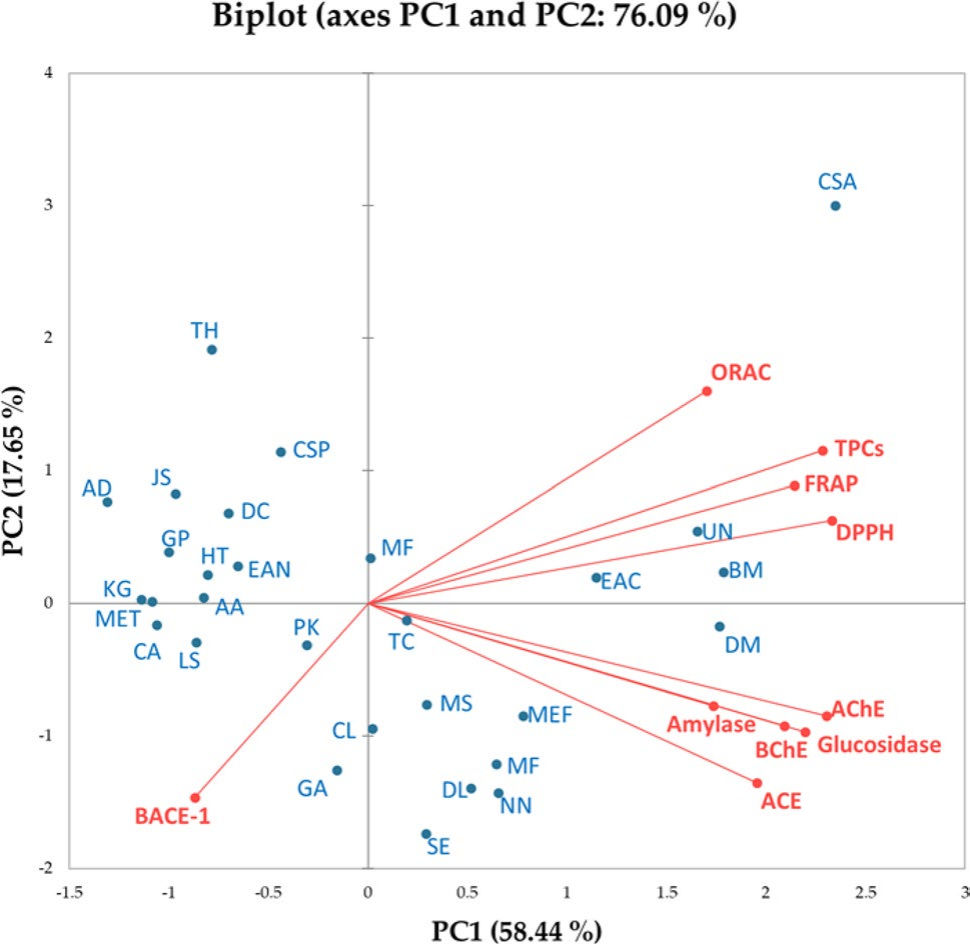
A biplot of principal component analysis (PCA) generated from the observations including aril (flesh), pericarp (peel) and seed of *Nephelium hypoleucum* Kurz and variables including total phenolic contents (TPCs), total anthocyanin contents (TACs), total flavonoid contents (TFCs), antioxidant activities (DPPH radical scavenging, FRAP and ORAC activities) and enzyme inhibitory activities against lipase, α-glucosidase, α-amylase, angiotensin-converting enzyme (ACE), β-secretase (BACE-1), butyrylcholinesterase (BChE) and acetylcholinesterase (AChE).

Principal component analysis (PCA) was used to further evaluate the data (Tables 7, 8). The PCA concept involves the reduction of large data sets into an interpretable figure while retaining as much of the original data as possible. To accomplish this, the mean values of TPCs, antioxidant activities (DPPP radical scavenging, FRAP, and ORAC activities), and enzyme inhibitory activities against key enzymes implicated in NCDs, such as α-amylase, α-glucosidase, AChE, BChE, BACE-1, and ACE were subjected to analysis. Data from Tables 7 and 8 were converted using PCA into a biplot, shown in Fig. 1, consisting of 2PCs including PC1 and PC2. The former covered 58.44% while the latter covered 17.65%, resulting in a total of 76.09% and indicating that the original data were represented and interpretable appropriately with minor error. PC1 contained α-amylase, α-glucosidase, AChE, BChE, and ACE inhibitory activities, while TPCs, antioxidant activities through DPPH radical scavenging, FRAP and ORAC assays, and BACE-1 inhibitory activities were positioned in PC2. Figure 1 illustrates the active variables (TPCs, antioxidant activities, and inhibitory activities against α-amylase, α-glucosidase, AChE, and BChE), while BACE-1 inhibitory activities were clustered together, implying some correlation between these variables. More TPCs were related to high antioxidant activity, as evaluated by the three assays. BACE-1 inhibitory activities were located opposite to other variables; thus, herbal samples with high BACE-1 activities tended to have low activities compared to other variables. Figure 1 also shows that most of the active observations (plant samples; such as TH, CSP, AD, JS, KG, MET, and CA) were clustered opposite the active variables, suggesting that they had low values in those variables. Interestingly, CA, GA, DL, and SE with high BACE-1 inhibitory activities were not located close to its position, implying that BACE-1 activities were not unique for these plants, while UN, EAC, BM, and DM were clustered with the most active variables. Therefore, except for BACE-1 activities, these four plants were high in TPCs, antioxidant activities, and enzyme inhibitory activities against key enzymes implicated in NCDs such as amylase, glucosidase, AChE, BChE, and ACE. Further investigations on the phytochemical profiles are required to unravel the bioactive compounds in these plants.

The results of antioxidant and enzyme inhibitory activities from the 29 herbal samples were complicated and difficult to interpret; consequently, two independent statistical analyses were used to overcome this hurdle. First, TOPSIS analysis was applied for multiple criteria decision-making, using the mean data of 10 dependent variables (TPCs, antioxidant activities determined by DPPP radical scavenging, FRAP and ORAC assays, and inhibitory activities against α-amylase, α-glucosidase, AChE, BChE, BACE-1, and ACE). The weight ratio for each variable was set at 0.1 as they were equally important. The positive ideal solution of Euclidean distance (D+), negative ideal solution of Euclidean distance (D−), and the closeness coefficient (C) of all 29 plant samples were determined. Among all the herbal samples, CSA exhibited the highest C score (0.827) determined from antioxidant activities by both HAT (ORAC assay) and SET (DPPH radical scavenging and FRAP assay) mechanisms (Supplementary Table 5). This result indicated that the CSA herb sample exhibited the highest antioxidant potential. Likewise, the BM herb sample showed the highest potential to inhibit the carbohydrate degrading enzymes α-amylase and α-glucosidase, with the highest C score of 0.993 (Supplementary Table 6). For the enzymes involved in Alzheimer’s disease (AChE, BChE, and BACE-1), DL exhibited the highest C score of 0.891 (Supplementary Table 7). Results in Table 8 showed that BM and DM enzymes involved in hypertension exhibited the highest ACE inhibitory activities. Overall, the plant with the highest C score was CSA (0.7588, Table 9), indicating that this sample had high TPC, antioxidant potential and enzyme-inhibiting activity. Conversely, AD (0.1337) harbored the lowest values of all tested variables. These statistical analyses demonstrate that among the 29 plant samples, CSA showed the highest potential in terms of TPC, antioxidant potential and enzyme-inhibiting activity.

**Table 9:** TOPSIS data and rank calculated from the mean data of ten dependent variables from 29 plant samples determined using overall activities.

Abbreviation	D+	D−	C	Rank
DL	0.103	0.092	0.4699	5
KG	0.132	0.030	0.1824	24
CSP	0.112	0.032	0.2222	18
DC	0.125	0.021	0.1455	27
CA	0.127	0.035	0.2178	19
AA	0.127	0.029	0.1873	22
GA	0.115	0.053	0.3146	11
MET	0.133	0.028	0.1718	26
SE	0.115	0.058	0.3358	10
MF	0.107	0.060	0.3608	8
CL	0.120	0.046	0.2767	14
NN	0.108	0.062	0.3648	7
TC	0.106	0.048	0.3122	12
GP	0.132	0.032	0.1936	21
TH	0.116	0.028	0.1956	20
AD	0.132	0.020	0.1337	29
JS	0.128	0.022	0.1446	28
EAN	0.124	0.028	0.1844	23
PK	0.120	0.042	0.2574	16
HT	0.129	0.028	0.1783	25
MF	0.111	0.041	0.2722	15
UN	0.071	0.084	0.5433	4
BM	0.070	0.090	0.5644	3
EAC	0.092	0.067	0.4200	6
MEF	0.108	0.059	0.3539	9
LS	0.117	0.040	0.2563	17
MS	0.117	0.049	0.2961	13
DM	0.074	0.103	0.5816	2
CSA	0.039	0.123	0.7588	1

List of abbreviation of samples are shown in Table 1. D+: positive ideal solution of Euclidean dis-tance; D−: negative ideal solution of Euclidean distance; C: the closeness coefficient. The weight ratio for each variable was equally set at 0.1.

## Discussion

Traditional herbs have been widely used as effective remedies to treat many diseases, from trivial symptoms like common fever to more severe infections such as COVID-19. Some of these green medicines are popular among locals due to their confidence in safe consumption and successful treatments. However, scant scientific-based evidence is available to support their medicinal properties. The health-related properties of each ingredient of PSCD and CHRT might also show promise for developing new remedies as other potential NCD treatments. This study investigated the herbal ingredients in the Thai traditional remedies PSCD and CHRT for their potential on reducing the risk of NCDs. Among all the herbal samples, TOPSIS analyses indicated that (i) CSA exhibited the highest overall results for TPC, antioxidant potential, and enzyme inhibition; (ii) BM possessed high potential to inhibit diabetes through α-amylase and α-glucosidase inhibition; (iii) BM and DM exhibited the highest ACE inhibition, leading to the control of hypertension; and (iv) DL retarded Alzheimer’s disease progression through cholinergic (AChE and BChE inhibitions) and β-amyloid formation (BACE-1 inhibition).

High TPCs in CSA gave high antioxidant potential in both HAT and SET mechanisms. A strong correlation between TPCs and antioxidant activities was also observed in rice, sacred lotus, and other plant extracts^12,16,23^, while water extract of CSA stem bark exhibited TPC of 682.67 mg GAE/g extract and DPPH radical scavenging activities with IC_50_ of 9.47 µg/mL as the highest among 16 galactogogue medicinal plants in Northeastern Thailand^24^. This TPC value corresponded to our 70% (*v/v*) aqueous ethanolic extract of CSA with TPC of 728.50 mg GAE/g extract. Due to its high TPC, the CSA extract also exhibited overall high enzyme inhibition determined by TOPSIS analysis. Sappan Lignum, extracted with 80% *(v/v*) aqueous ethanol, exhibited α-glucosidase inhibitory activity with IC_50_ of 6.3 µg/mL^25^, while an in vivo experiment indicated that CSA exhibited antidyslipidemic and antidiabetic activities in alloxan induced diabetic rat^26^. No previous studies have reported on the anti-Alzheimer’s disease property of CSA, and this study is the first to report its anti-Alzheimer’s disease potential through the inhibition of cholinesterases and BACE-1 activities. The phenolic profile analyzed by LC–ESI–MS/MS indicated that CSA exhibited predominantly chlorogenic acid, followed by 3,4-dihydroxybenzoic acid and a trace of naringenin. CSA exhibited high TPC but its phenolic profile indicated moderate to low phenolic content, suggesting that CSA contained particular phenolics other than the common ones used as our LC–ESI–MS/MS standards. Previous research on phenolic profiles of CSA analyzed by liquid chromatography-quadrupole time-of-flight tandem mass spectrometry (LC–Q-TOF–MS/MS) found 4,7 dihydroxycoumarin as a major compound (24.47% area sum) with methyl 7-desoxypurpurogallin-7-carboxylate trimethyl ether, brazilein, and biochanin A (approximately (8% area sum) as the minor compounds^27^. These compounds added up to high TPC distribution in CSA. No previous report has been published on enzyme inhibitory activities of 4,7 dihydroxycoumarin, with previous research mostly focusing on 7,8 dihydroxycoumarin (esculetin). Esculetin at 500 μg/mL inhibited AChE at 97%^28^, while giving half-maximal inhibitory concentration (IC_50_) at 69 µM against α-glucosidase^29^. This compound also prevented hypertension in insulin resistant and type 2 diabetic rats^30^. An in-silico analysis suggested that dihydroxy coumarin interacted with the peroxisome proliferator-activated receptor (PPAR) but with lower binding affinity than orlistat (a competitive lipase inhibitor)^31^. These reports suggested that 4,7 dihydroxycoumarin might also exhibit these properties with different degrees of activity. Other bioactive compounds in CSA were previously reported for their health-related properties. A comprehensive pharmacologic investigation suggested that fisetin tetramethyl ether could be responsible for T2DM management through T2DM associated signaling pathways and target receptors including proliferator-activated receptor gamma (PPARG)^32^. Other bioactive compounds in CSA including brazilin, sappanchalcone and protosappanins A-E, along with its methanolic extract, possessed vasorelaxant activities on rat aorta and the mesenteric artery^33^.

BM strongly inhibited α-amylase and α-glucosidase, the key enzymes in T2DM control. Many plant parts including fruit, seed, stem, and leaves have been previously reported for BM^34^ but no reports are available on its root. Thus, this is the first report on the phenolic profile and health-related properties of BM root. BM exhibited the second most abundant TPCs, with high levels of phenolic acids including gallic acid and 3,4-dihydioxybenzoic acid, moderate amounts of flavonoids including luteolin and naringenin detected by LC–ESI–MS/MS. As the most abundant phenolic detected in BM, gallic acid inhibited α-amylase with IC_50_ ranging 4.35–13.69 mM depending on the assay method^35^, while another study indicated that 3,4-dihydroxybenzoic acid exhibited a similar IC_50_ value to gallic acid^36^. When compared to acarbose, a commercially available T2DM drug acting as an α-amylase and α-glucosidase competitive inhibitor, gallic acid and 3,4-dihydroxybenzoic acid were less effective, with IC_50_ ranging 0.018–0.300 mM against α-amylase^35^. The inhibitory effect of gallic acid was also observed in α-glucosidase assay, inhibiting α-glucosidase in a competitive manner with a 2.4-fold higher IC_50_ value than acarbose (15.87 µM)^37^. However, 3,4-dihydroxybenzoic acid exhibited IC_50_ of 3.64 mM against α-glucosidase^38^, suggesting that this phenolic inhibited α-glucosidase at a lower strength than gallic acid.

Two herbal extracts, BM and DM, strongly inhibited ACE, the key enzyme contributing to hypertension. Similar to BM, DM also exhibited high TPCs with gallic acid and 3,4-dihydroxybenzoic acid abundantly detected. A recent ethnopharmacological database only indicated traditional usage of DM^39^ but its methanolic extract (unknown plant part) at a concentration of 1 mg/mL was unable to inhibit ACE^40^. At the same concentration, our aqueous ethanolic extract of DM exhibited 81.19% inhibition against ACE. As the most abundant phenolics, gallic acid exhibited IC_50_ of 0.33 mM against ACE, while 3,4-dihydroxybenzoic acid exhibited < 50% inhibition at a concentration of 0.5 mM^41^. However, these phenolics exhibited lower ACE inhibitory strength than the commercially available ACE inhibitors captopril (IC_50_ 15.85–79.40 nM), lisinopril (IC_50_ 5.62–31.6 nM), and enalapril (IC_50_ 1.2–70.0 µM) (IC_50_ values varied depending on the tissues being tested)^42^.

Likewise, DL strongly inhibited cholinergic (AChE and BChE) and β-amyloid formation enzymes (BACE-1), thereby showing potential as an effective agent to control Alzheimer’s disease. Interestingly, DL or Chinese Dragon’s Blood has long been reported to exhibit neuroprotective effects through its bioactive compound, loureirin C^43^. Loureirin C was recently shown to improve cognitive impairment in Alzheimer’s disease genetically modified mice via estrogen receptor α (ERα)^44^, while stemwood extract of DL prevented amyloid-β (Aβ) fibril formation and disassembled Aβ aggregation, leading to reduced Aβ-induced neuronal toxicity^7^. Several bioactive compounds were detected in DL including resveratrol, pterostilbene, loureirin B, and loureirin A that were able to prevent Aβ fibril-induced cell death; however, these compounds provided a weaker effect than crude DL extract^7^. Thus, the synergistic effect or other bioactive compounds might be responsible for this activity. Our LC–ESI–MS/MS analysis indicated that DL exhibited high apigenin content with a moderate amount of naringenin, while no phenolic acid was detected in this herbal extract. Apigenin (25–100 µM) improved climbing ability in a transgenic *Drosophila* model of Alzheimer’s disease, with similar results to the 0.1 mM donepezil treated group compared to unexposed AD flies^45^. The AD flies exposed to apigenin (50–100 µM) exhibited lower AChE activities and formation of Aβ aggregation than the unexposed AD flies^45^. A molecular docking investigation indicated AChE structural change due to the binding of apigenin at the peripheral binding site (flexible part that locates near the entrance of the AChE active site), indicating a noncompetitive type of inhibitor^46^. Thus, apigenin could be another bioactive compound in DL contributing to the retardment of Alzheimer’s disease development.

## Conclusion

Twenty-nine herb ingredients in two Thai traditional remedies, PSCD and CHRT, which are famous for their fever-lowering properties, were investigated for their phenolic profiles, antioxidant potentials, and inhibition of the key enzymes controlling the risk of NCDs. CSA exhibited the highest overall results on TPCs, antioxidant potentials, and all enzyme inhibitions. However, when considering each particular NCD, BM possessed high potential to control T2DM occurrence through inhibition of α-amylase and α-glucosidase, while BM and DM showed potential to ameliorate hypertension through ACE inhibition. DL controlled Alzheimer’s disease progression through the cholinergic enzymes (AChE and BChE) and the β-amyloid forming enzyme (BACE-1). This preliminary information can be used to develop new herbal remedies or extracts for the prevention or treatment of NCDs.

### Supplementary Information

Supplementary Information.

## Data Availability

All data generated or analyzed during this study are included in this published article and its Supplementary Information.
