# Supplementary material for: Phenolic profiles and in vitro biochemical properties of Thai herb ingredients for chronic diseases prevention
Source: Sci Rep. 2023 Dec 7;13:21690. doi: 10.1038/s41598-023-49074-5 (PMC10709644; doi:10.1038/s41598-023-49074-5)

Supplementary Material

Contents

Tables

Table 1: The combination of ingredients in two Thai traditional remedies including Prasachandaeng (PSCD) and Chantaharuethai (CHRT).	*1*
Table 2: The physical appearance of each ingredient in two Thai traditional remedies including Prasachandaeng (PSCD) and Chantaharuethai (CHRT).	*3*
Table 3: The validation parameters of twenty-four authentic standards of phenolics using LC-ESI-MS/MS in selective reaction monitoring (SRM) mode.	*13*
Table 4: Raw data of three antioxidant activities determined by 2,2-diphenyl-1-picrylhydrazyl (DPPH) radical scavenging, ferric ion reducing antioxidant power (FRAP) and oxygen radical absorbance capacity (ORAC) assays of twenty-nine herb samples.	*14*
Table 5: TOPSIS data and rank calculated from the mean data of three antioxidant activities determined by 2,2-diphenyl-1-picrylhydrazyl (DPPH) radical scavenging, ferric ion reducing antioxidant power (FRAP) and oxygen radical absorbance capacity (ORAC) assays of twenty-nine herb samples.	*15*
Table 6: TOPSIS data and rank calculated from the mean inhibitory data of two carbohydrate degrading enzymes including α-amylase and α-glucosidase of twenty-nine herb samples.	*16*
Table 7: TOPSIS data and rank calculated from the mean inhibitory data of three enzymes involved in Alzheimer's disease occurrence including acetylcholinesterase (AChE), butyrylcholinesterase (BChE) and β-secretase (BACE-1) of twenty-nine herb samples.	*17*

Figures

Figure 1: The liquid chromatography-electrospray ionization tandem mass spectrometry (LC-ESI-MS/MS) chromatograms of the herb extracts.	*18*

Supplementary

Table 1 The combination of ingredients in two Thai traditional remedies including Prasachandaeng (PSCD) and Chantaharuethai (CHRT).

Prasachandaeng (PSCD) Ingredients		
**Scientific Name**	**Plant Part**	**Weight (Total of 64 g)**
Dracaena cochinchinensis (Lour.) S.C. Chen	Heartwood	32 g
Ligusticum sinense Oliv.	Rhizome	4 g
Myristica fragrans Houtt.	Heartwood	4 g
Kaempferia galanga L.	Rhizome	4 g
Biancaea sappan (L.) Tod.	Heartwood	4 g
Citrus x aurantifolia (Christm.) Swingle	Root	4 g
Bouea macrophylla Griff.	Root	4 g
Heliciopsis terminalis (Kurz) Sleumer	Root	4 g
Nelumbo nucifera Gaertn.	Stamen	1 g
Mesua ferrea L.	Flower	1 g
Jasminum sambac (L.) Aiton	Flower	1 g
Mammea siamensis (Miq.) T. Anderson	Flower	1 g

Supplementary

Table 1 The combination of ingredients in two Thai traditional remedies including Prasachandaeng (PSCD) and Chantaharuethai (CHRT) (Cont.)

Chantaharuethai (CHRT) Ingredients		
**Scientific Name**	**Plant Part**	**Weight (Total of 277.5 g)**
Dracaena cochinchinensis (Lour.) S.C. Chen	Heartwood	15 g
Tarenna hoaensis Pit.	Heartwood	15 g
Myristica fragrans Houtt.	Heartwood	15 g
Euphorbia antiquorum L.	Heartwood	15 g
Mimusops elengi L.	Heartwood*	15 g
Urceola minutiflora (Pierre) D.J. Middleton	Stem	15 g
Aristolochia pierrei Lecomte	Root	15 g
Carissa spinarum L.	Heartwood	15 g
Gynura pseudochina (L.) DC.	Rhizome	15 g
Glycyrrhiza glabra L.	Root	15 g
Tacca chantrieri André	Whole plant	15 g
Calamus longisetus Griff.	Stem	15 g
Angelica dahurica (Hoffm.) Benth. & Hook.f. ex Franch. & Sav.	Root	7.5 g
Artemisia annua L.	Root	7.5 g
Dischidia major (Vahl) Merr.	Root	7.5 g
Picrorhiza kurroa Royle ex Benth.	Rhizome	7.5 g
Ligusticum sinense Oliv.	Rhizome	7.5 g
Mimusops elengi L.	Flower	7.5 g
Mesua ferrea L.	Flower	7.5 g
Mammea siamensis (Miq.) T. Anderson	Flower	7.5 g
Jasminum sambac (L.) Aiton	Flower	7.5 g
Nelumbo nucifera Gaertn.	Stamen	7.5 g
Dendrobium crumenatum Sw.	Stem	7.5 g
Sophora exigua Craib	Root	7.5 g
Enhalus acoroides (L.f.) Royle	Rhizome	3.75 g
Hibiscus surattensis L.	-	1.875 g
Pogostemon cablin	-	1.875 g

Three ingredients in CHRT including *Aristolochia pierrei* Lecomte, *Hibiscus surattensis* L. (musk), and *Pogostemon cablin* (Borneo camphor) were not investigated because the first was previously reported for its toxicity. * Rotten wood.

Supplementary

Table 2 The physical appearance of each ingredient in two Thai traditional remedies including Prasachandaeng (PSCD) and Chantaharuethai (CHRT).

Scientific Names	Abbreviation	Plant Parts	Physical appearance
Jasminum sambac (L.) Aiton	JS	Flower	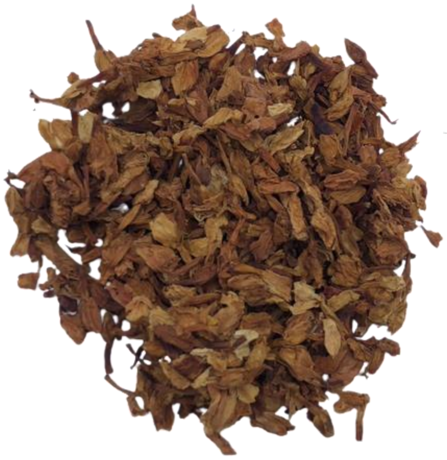
Mammea siamensis (Miq.) T. Anderson	MS	Flower	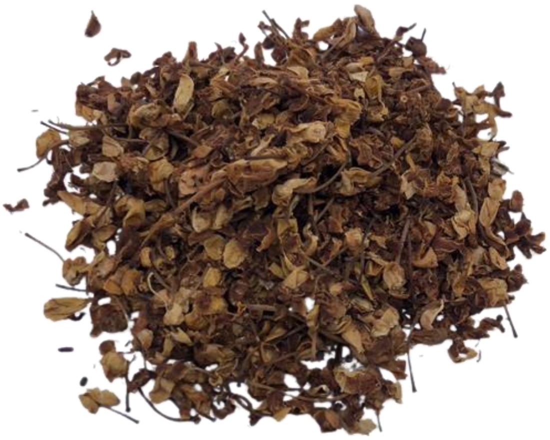
Mesua ferrea L.	MF	Flower	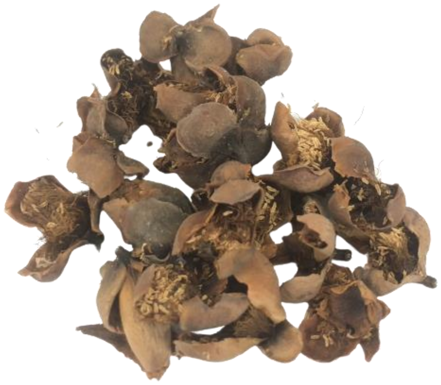

Supplementary

**Scientific Names**	**Abbreviation**	**Plant Parts**	**Physical appearance**
Mimusops elengi L.	MEF	Flower	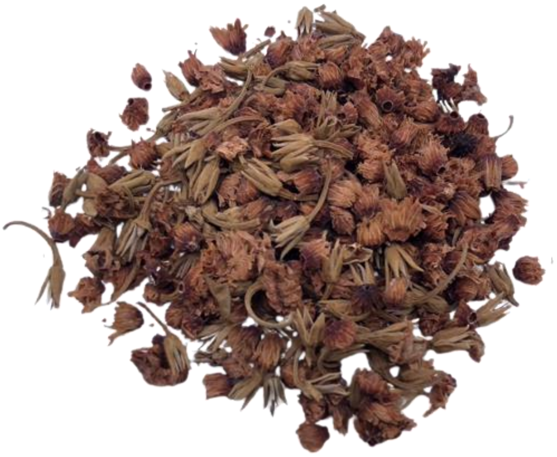
Nelumbo nucifera Gaertn.	NN	Stamen	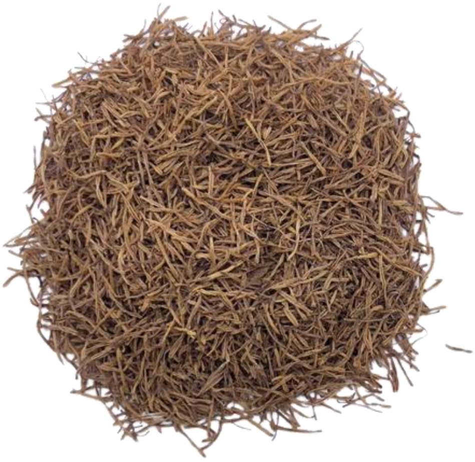
Calamus longisetus Griff.	CL	Stem	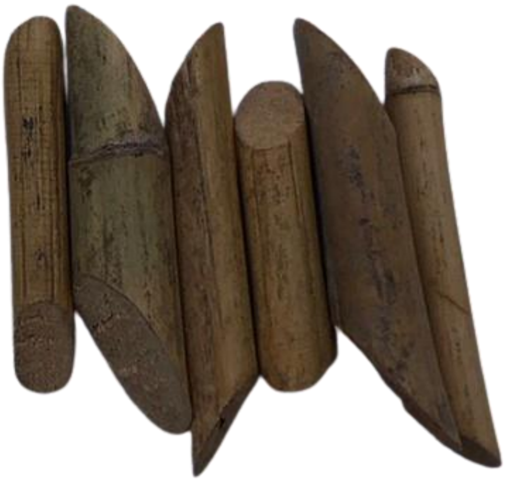

Supplementary

**Scientific Names**	**Abbreviation**	**Plant Parts**	**Physical appearance**
Dendrobium crumenatum Sw.	DC	Stem	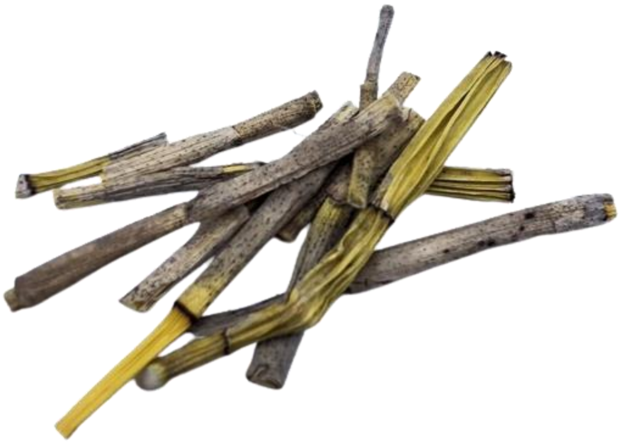
Urceola minutiflora (Pierre) D.J. Middleton	UN	Stem	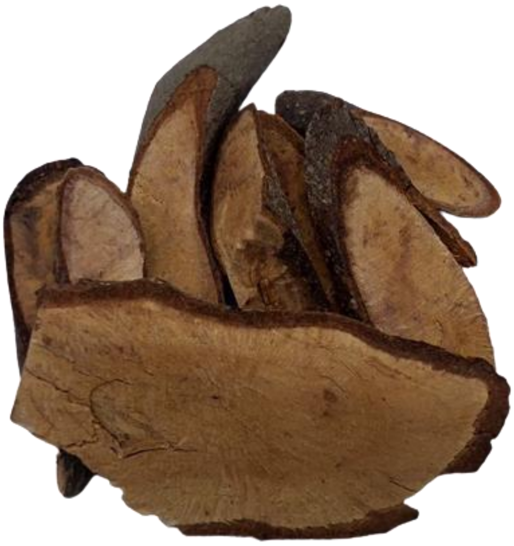
Tacca chantrieri André	TC	Whole plant	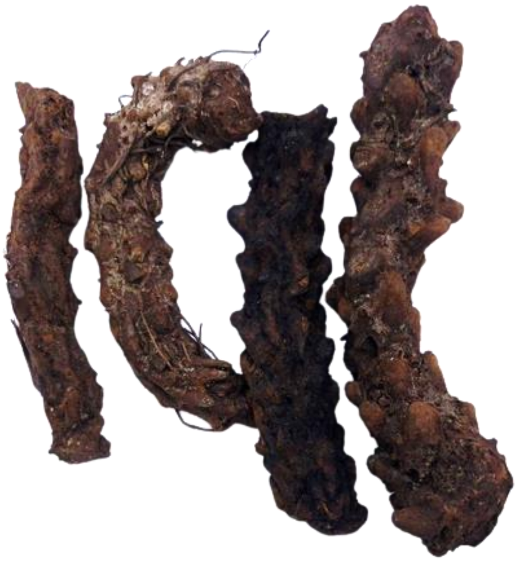

Supplementary

Scientific Names	Abbreviation	Plant Parts	Physical appearance
Biancaea sappan (L.) Tod.	CSA	Heartwoo d	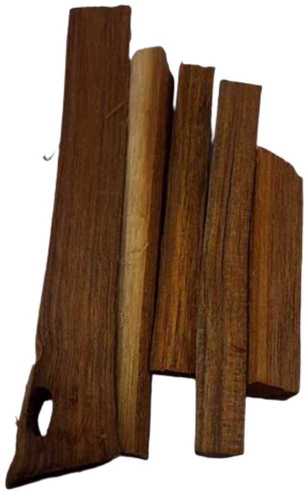
Carissa spinarum L.	CSP	Heartwoo d	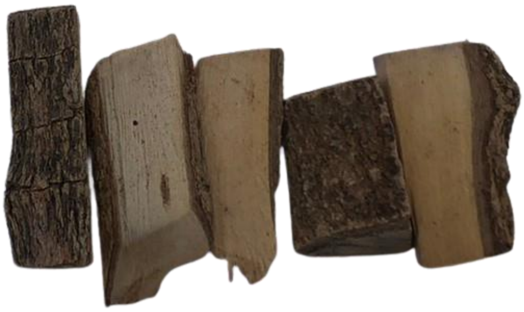
Dracaena cochinchinensis (Lour.) S.C. Chen	DL	Heartwoo d	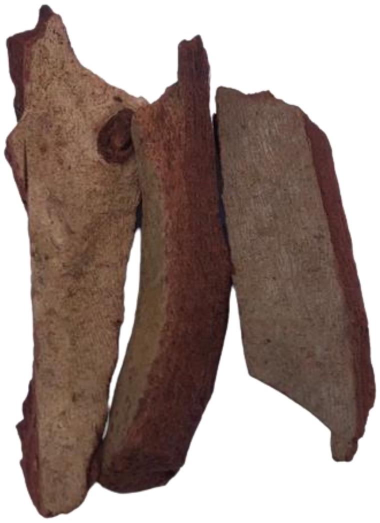

Supplementary

Scientific Names	Abbreviation	Plant Parts	Physical appearance
Euphorbia antiquorum L.	EAN	Heartwoo d	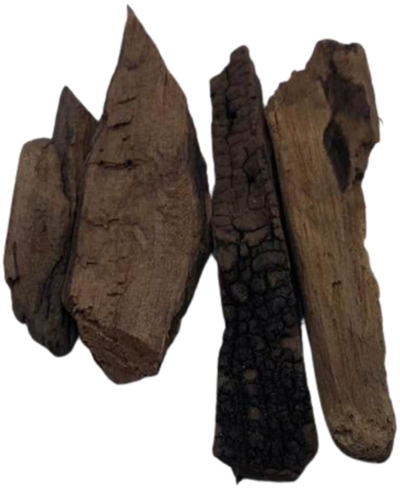
Mimusops elengi L.	MET	Heartwoo d *	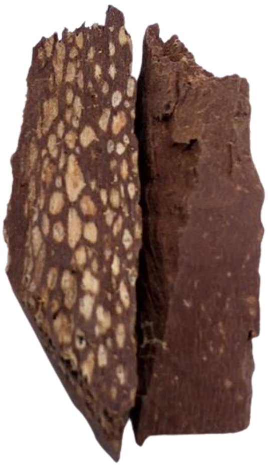
Myristica fragrans Houtt.	MF	Heartwoo d	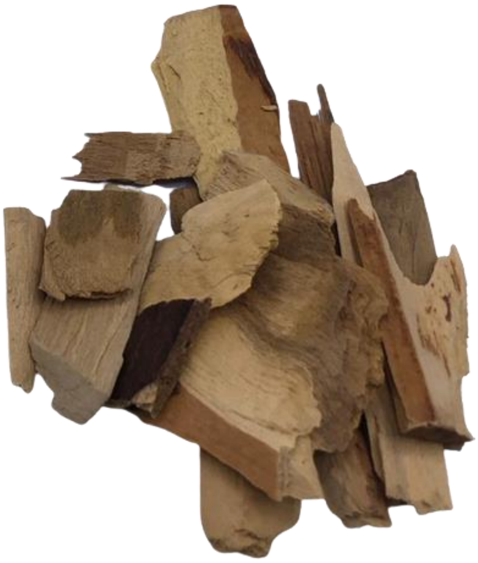

Supplementary

Scientific Names	Abbreviation	Plant Parts	Physical appearance
Tarenna hoaensis Pit.	TH	Heartwood	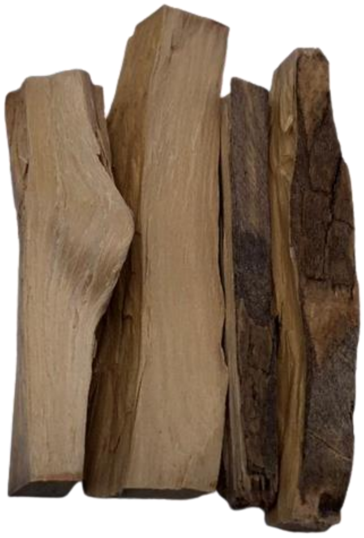
Enhalus acoroides (L.f.) Royle	EAC	Rhizome	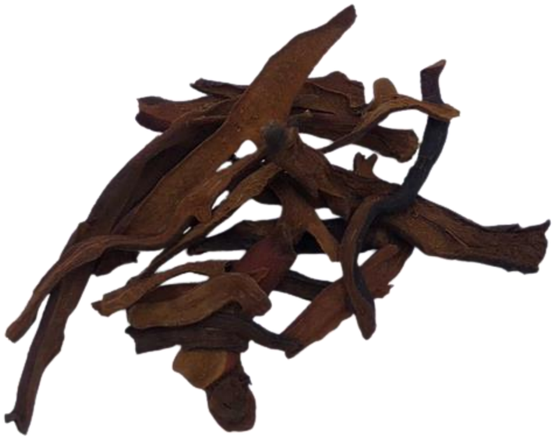
Gynura pseudochina (L.) DC.	GP	Rhizome	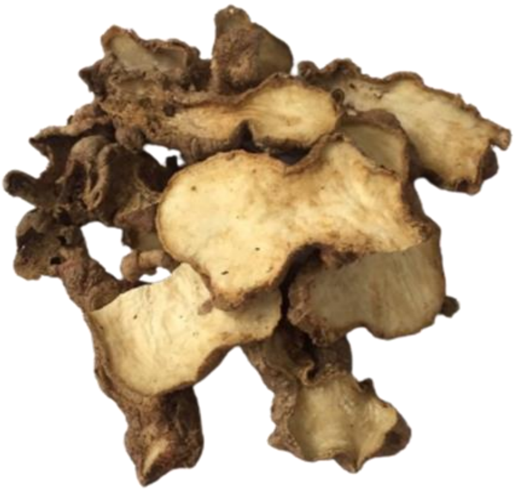

Supplementary

**Scientific Names**	**Abbreviation**	**Plant Parts**	**Physical appearance**
Kaempferia galanga L.	KG	Rhizome	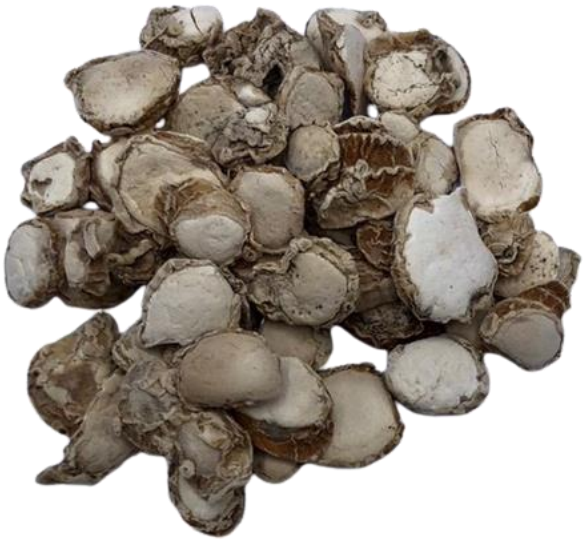
Ligusticum sinense Oliv.	LS	Rhizome	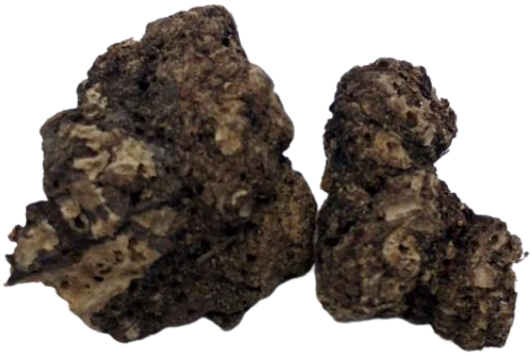
Picrorhiza kurroa Royle ex Benth.	PK	Rhizome	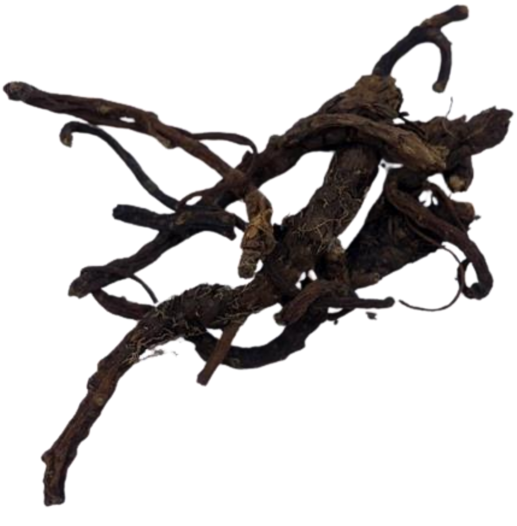

Supplementary

Scientific Names	Abbreviation	Plant Parts	Physical appearance
Angelica dahurica (Hoffm.) Benth. & Hook.f. ex Franch. & Sav.	AD	Root	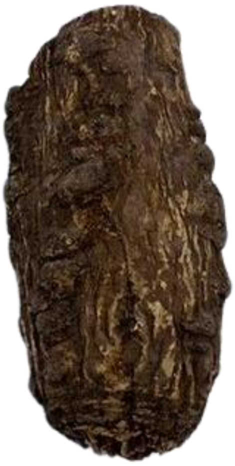
Artemisia annua L.	AA	Root	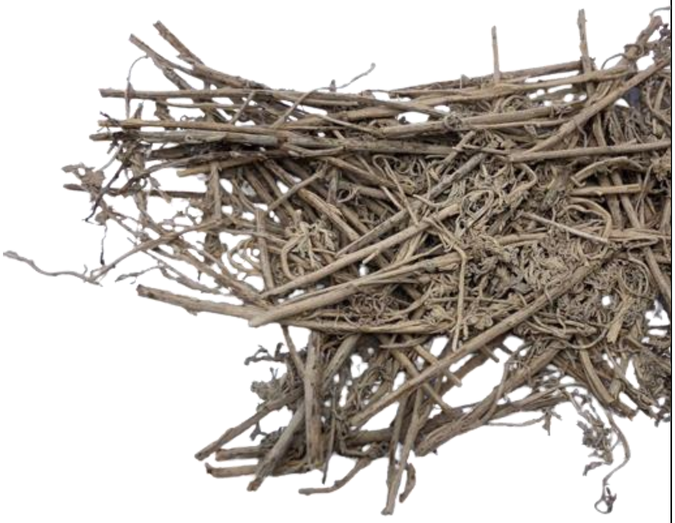
Bouea macrophylla Griff.	BM	Root	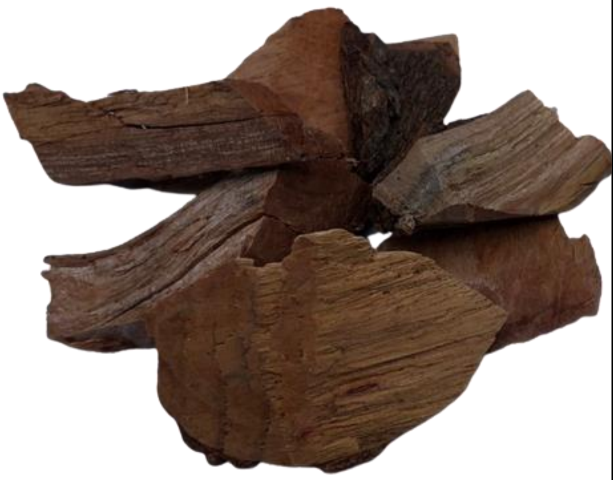

Supplementary

**Scientific Names**	**Abbreviation**	**Plant Parts**	**Physical appearance**
Citrus x aurantifolia (Christm.) Swingle	CA	Root	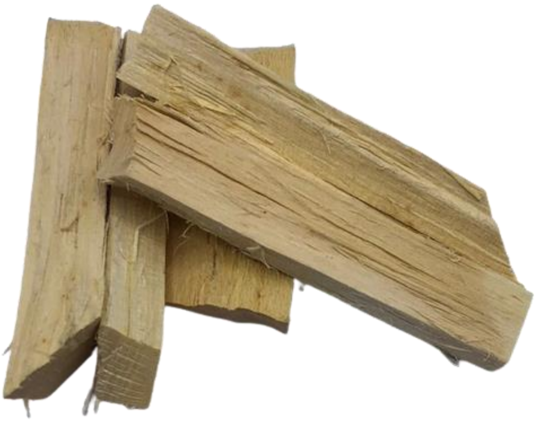
Dischidia major (Vahl) Merr.	DM	Root	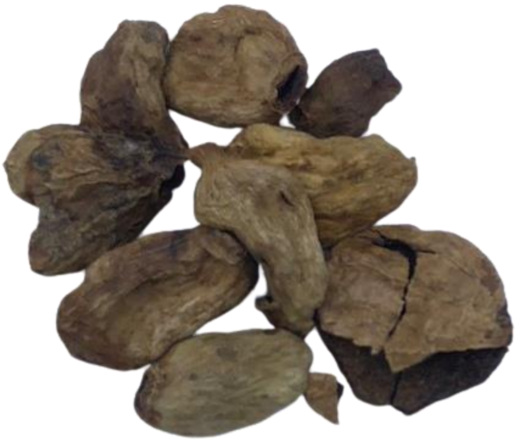
Glycyrrhiza glabra L.	GA	Root	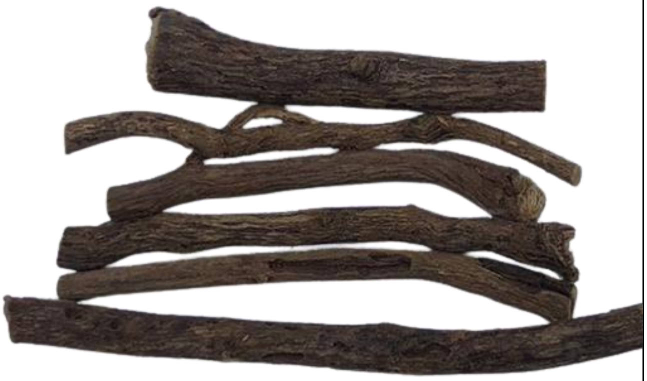

Supplementary

Scientific Names	Abbreviation	Plant Parts	Physical appearance
Heliciopsis terminalis (Kurz) Sleumer	HT	Root	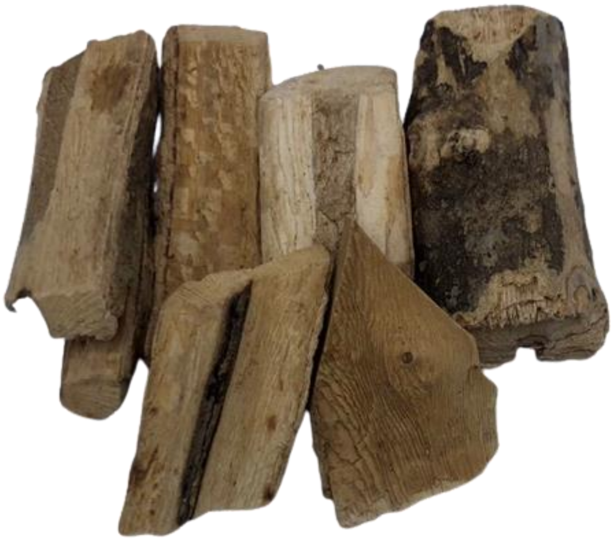
Sophora exigua Craib	SE	Root	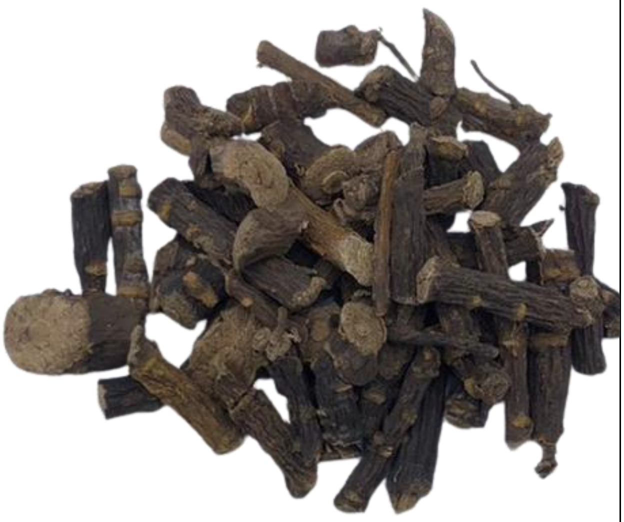

Supplementary

Table 3: The validation parameters of twenty-four authentic standards of phenolics using LC-ESI-MS/MS in selective reaction monitoring (SRM) mode.*

Compounds	Retention time (min)	Standards	Linear range (µg/mL)	Linear regression equation	Correlation coefficient (R^2)	LOD (µg/mL)	LOQ (µg/mL)	%RSD (Inter-day)	%Recovery		
									Low level (µg/mL)	Medium level (µg/mL)	High level (µg/mL)
1	0.44	Epigallocatechin gallate	0.125–40	y =8533x + 1053.4	0.9985	0.067	0.230	0.023	91.84	85.36	91.37
2	0.564	Gallic acid	0.195–25	y =3323.1x – 2100.4	0.9984	0.04	0.14	0.01	113.05	118.57	109.12
3	0.803	3,4-Dihydroxybenzoic acid	0.195–25	y =11490x – 10877	0.9935	0.010	0.034	0.003	90.59	85.75	89.75
4	0.922	Chlorogenic acid	0.3125–40	y =8377.5x – 3623.5	0.9934	0.017	0.055	0.006	91.94	87.50	95.02
5	1.16	4-Hydroxybenic acid	0.3125–40	y =2482.6x– 3998.4	0.9917	0.027	0.090	0.009	109.67	103.60	101.28
6	1.40	Caffeic acid	0.3125–40	y =12328x–19725	0.9918	0.010	0.035	0.003	105.36	93.98	87.41
7	1.539	Syringic acid	3.125–100	y =68.091x + 230.43	0.9955	0.582	1.939	0.194	116.35	97.42	94.91
8	1.63	Vanillic acid	2.5–100	y =213.67x – 975.72	0.9900	0.15	0.48	0.05	99.86	101.76	100.12
9	2.452	*p*-Coumaric acid	0.3125–40	y =8532.4x–13559	0.9910	0.013	0.042	0.004	88.22	81.36	98.05
10	2.737	Rutin	0.009–1.25	y =49729x – 33.064	0.9999	0.001	0.005	0.0005	94.63	114.00	108.73
11	2.772	Sinapic acid	0.39–25	y =1592.6x – 832.22	0.9977	0.026	0.086	0.009	81.34	92.16	84.22
12	2.851	Ferulic acid	1.56–100	y =559.03x – 1819.2	0.9947	0.155	0.518	0.052	91.51	89.24	93.10
13	3.41	Hesperidin	0.25–40	y =838.63x – 242.2	0.9986	0.07	0.22	0.02	100.43	104.06	108.60
14	3.431	Myricetin	1.25–40	y =303.47x – 601.81	0.9976	0.261	0.871	0.087	113.07	81.77	91.12
15	3.528	Rosmarinic acid	0.3125–40	y =4322.4x-3744.1	0.9956	0.07	0.25	0.02	92.45	106.35	99.62
16	4.158	Luteolin	0.195–12.5	y =8381.9x – 5000.7	0.9945	0.015	0.050	0.0005	84.21	96.21	107.09
17	4.185	Quercetin	0.05–12.5	y =2934x + 917.17	0.9937	0.05	0.18	0.02	83.36	115.06	95.74
18	4.522	Cinnamic acid	0.039–10	y =6631.9x – 866.59	0.9964	0.049	0.163	0.016	101.94	98.84	95.85
19	4.689	Apigenin	0.34–11	y =1790.7x – 287.7	0.9997	0.127	0.424	0.042	88.84	106.89	114.79
20	4.693	Genistein	0.625–40	y =1247.2x – 1747.1	0.9977	0.049	0.163	0.016	95.33	101.49	11633
21	4.705	Naringenin	0.0008–5	y =16755x + 443.03	0.9932	0.003	0.011	0.001	117.92	96.26	111.08
22	4.79	Kaempferol	0.25–10	y =1006.8x – 346.28	0.9905	0.122	0.406	0.041	92.35	107.69	102.17
23	4.878	Isorhamnetin	0.0098–2.5	y =12698x + 586.16	0.9945	0.016	0.052	0.005	113.57	105.88	111.14
24	6.146	Galangin	0.3125–40	y =5012.1x – 9354.7	0.9879	0.010	0.035	0.003	84.01	112.92	115.80

*These data are from our previous study (Sirichai et al., 2022)[16].

Supplementary

Table 4 Raw data of three antioxidant activities determined by 2,2-diphenyl-1-picrylhydrazyl (DPPH) radical scavenging, ferric ion reducing antioxidant power (FRAP) and oxygen radical absorbance capacity (ORAC) assays of twenty-nine herb samples.

**Abbreviation**	DPPH radical scavenging activities (μmol TE/g)			FRAP activities (μmol TE/g)			ORAC activities (μmol TE/g)		
	N1	N2	N3	N1	N2	N3	N1	N2	N3
DL	0.57	0.57	0.55	539.09	560.91	530.00	3912.16	3507.49	3487.03
KG	0.14	0.13	0.15	201.96	196.52	172.17	342.81	330.77	335.92
CSP	0.67	0.64	0.64	1023.04	956.09	940.87	3287.28	3069.25	3000.94
DC	0.32	0.34	0.30	302.17	343.48	318.91	1704.54	1836.08	2072.56
CA	0.18	0.20	0.19	327.39	330.87	324.13	1511.96	1214.18	1407.24
AA	0.27	0.27	0.26	274.78	280.22	274.13	1093.01	1048.08	1112.91
GA	0.30	0.30	0.30	164.55	181.36	185.91	1923.94	1904.04	2095.23
MET	0.16	0.16	0.17	120.00	133.91	129.13	230.21	242.99	259.61
SE	0.34	0.33	0.34	249.77	255.23	255.91	884.58	792.84	898.08
MF	0.72	0.75	0.75	523.41	529.55	545.00	2181.06	2204.07	2368.43
CL	0.41	0.37	0.40	285.68	299.55	298.41	940.47	931.91	949.24
NN	0.67	0.68	0.70	1027.73	1005.00	1025.00	1011.51	1063.73	1102.08
TC	0.56	0.57	0.61	737.27	765.00	744.55	3443.36	3148.28	3028.74
GP	0.12	0.12	0.12	129.13	137.83	138.26	345.38	335.39	300.89
TH	0.50	0.50	0.52	981.82	1039.09	968.18	3967.58	3965.59	4458.84
AD	0.15	0.15	0.15	151.96	160.00	156.74	1151.85	980.90	1023.26
JS	0.32	0.32	0.33	275.23	266.59	260.68	1503.58	1528.61	1508.50
EAN	0.49	0.49	0.49	411.36	408.64	396.36	1097.29	944.96	1080.17
PK	0.31	0.31	0.31	318.41	302.73	305.00	1761.60	1762.51	1712.47
HT	0.20	0.21	0.19	306.74	306.74	328.91	767.62	687.66	738.17
MF	0.78	0.66	0.69	933.48	968.70	968.83	2911.21	2992.72	2949.82
UN	2.04	1.80	2.12	2698.26	2767.83	2761.74	4996.89	5177.89	5673.83
BM	2.03	1.83	1.82	3125.22	3160.00	3291.30	3919.40	3885.08	4306.29
EAC	0.80	0.80	0.94	1376.82	1404.09	1395.00	4894.51	4944.15	4693.16
MEF	0.96	0.91	0.83	970.87	959.13	947.83	1135.29	1256.41	1364.96
LS	0.17	0.20	0.19	131.74	126.74	127.83	623.61	637.51	581.03
MS	0.56	0.53	0.52	623.91	631.74	635.22	1145.61	1219.71	1302.00
DM	2.51	2.25	2.28	5195.22	5268.26	5127.39	2373.77	2319.55	2034.06
CSA	1.97	1.73	1.71	3831.30	3943.48	3734.78	16582.55	15135.78	16002.27

Supplementary

Table 5 TOPSIS data and rank calculated from the mean data of three antioxidant activities determined by 2,2-diphenyl-1-picrylhydrazyl (DPPH) radical scavenging, ferric ion reducing antioxidant power (FRAP) and oxygen radical absorbance capacity (ORAC) assays of twenty-nine herb samples.

Abbreviation	D+	D-	C	Rank
DL	0.299	0.066	0.180	9
KG	0.357	0.003	0.009	28
CSP	0.292	0.069	0.190	8
DC	0.331	0.031	0.086	15
CA	0.340	0.021	0.057	21
AA	0.343	0.018	0.050	23
GA	0.334	0.031	0.085	17
MET	0.359	0.004	0.010	27
SE	0.344	0.019	0.053	22
MF	0.310	0.057	0.155	12
CL	0.340	0.023	0.064	20
NN	0.315	0.055	0.149	13
TC	0.298	0.063	0.174	11
GP	0.359	0.001	0.004	29
TH	0.285	0.077	0.213	6
AD	0.349	0.013	0.037	24
JS	0.336	0.026	0.072	19
EAN	0.334	0.031	0.086	16
PK	0.333	0.029	0.080	18
HT	0.348	0.012	0.034	25
MF	0.293	0.069	0.191	7
UN	0.200	0.186	0.483	3
BM	0.211	0.185	0.468	4
EAC	0.257	0.104	0.287	5
MEF	0.308	0.066	0.177	10
LS	0.354	0.008	0.022	26
MS	0.325	0.039	0.107	14
DM	0.221	0.258	0.538	2
CSA	0.066	0.317	0.827	1

List of abbreviation of samples are shown in Table 1. (D+); positive ideal solution of Euclidean distance, (D-) negative ideal solution of Euclidean distance and (C): the closeness coefficient.

Table 6 TOPSIS data and rank calculated from the mean inhibitory data of two carbohydrate degrading enzymes including α-amylase and α-glucosidase of twenty-nine herb samples.

**Abbreviation**	**D+**	**D-**	**C**	**Rank**
DL	0.012	0.196	0.943	4
KG	0.175	0.054	0.237	21
CSP	0.156	0.058	0.271	20
DC	0.182	0.040	0.181	24
CA	0.183	0.039	0.174	25
AA	0.173	0.042	0.197	22
GA	0.082	0.131	0.614	12
MET	0.181	0.041	0.184	23
SE	0.045	0.165	0.786	9
MF	0.029	0.181	0.863	8
CL	0.079	0.151	0.657	11
NN	0.009	0.199	0.955	3
TC	0.095	0.122	0.562	14
GP	0.131	0.142	0.520	15
TH	0.206	0.000	0.000	29
AD	0.176	0.031	0.150	27
JS	0.202	0.007	0.032	28
EAN	0.167	0.073	0.303	19
PK	0.152	0.089	0.371	17
HT	0.182	0.033	0.155	26
MF	0.163	0.081	0.332	18
UN	0.059	0.163	0.733	10
BM	0.001	0.205	0.993	1
EAC	0.005	0.203	0.978	2
MEF	0.021	0.185	0.899	6
LS	0.117	0.151	0.562	13
MS	0.155	0.117	0.429	16
DM	0.019	0.191	0.911	5
CSA	0.022	0.188	0.893	7

List of abbreviation of samples are shown in Table 1. (D+); positive ideal solution of Euclidean distance, (D-) negative ideal solution of Euclidean distance and (C): the closeness coefficient.

Table 7 TOPSIS data and rank calculated from the mean inhibitory data of three enzymes involved in Alzheimer's disease occurrence including acetylcholinesterase (AChE), butyrylcholinesterase (BChE) and β-secretase (BACE-1) of twenty-nine herb samples.

Abbreviation	D+	D-	C	Rank
DL	0.014	0.111	0.891	1
KG	0.079	0.118	0.598	9
CSP	0.091	0.100	0.525	26
DC	0.098	0.103	0.512	29
CA	0.075	0.131	0.636	7
AA	0.062	0.099	0.615	8
GA	0.037	0.114	0.755	4
MET	0.105	0.127	0.548	17
SE	0.017	0.106	0.861	2
MF	0.049	0.095	0.661	6
CL	0.065	0.088	0.575	15
NN	0.039	0.096	0.712	5
TC	0.081	0.093	0.535	23
GP	0.119	0.128	0.520	27
TH	0.122	0.128	0.513	28
AD	0.110	0.130	0.541	20
JS	0.085	0.098	0.535	22
EAN	0.079	0.096	0.546	18
PK	0.064	0.099	0.608	9
HT	0.062	0.091	0.594	12
MF	0.087	0.099	0.533	24
UN	0.077	0.101	0.565	16
BM	0.065	0.096	0.596	11
EAC	0.107	0.119	0.526	25
MEF	0.084	0.100	0.543	19
LS	0.078	0.113	0.591	13
MS	0.067	0.096	0.588	14
DM	0.023	0.106	0.823	3
CSA	0.096	0.112	0.538	21

List of abbreviation of samples are shown in Table 1. (D+); positive ideal solution of Euclidean distance, (D-) negative ideal solution of Euclidean distance and (C): the closeness coefficient.

Supplementary

Figure 1 The liquid chromatography-electrospray ionization tandem mass spectrometry (LC-ESI-MS/MS) chromatograms of the herb extracts including (A) Jasminum sambac (L.) Aiton, (B) Mammea siamensis (Miq.) T. Anderson, (C) Mesua ferrea L., (D) Mimusops elengi L. (flower), (E) Nelumbo nucifera Gaertn., (F) Calamus longisetus Griff., (G) Dendrobium crumenatum Sw., (H) Urceola minutiflora (Pierre) D.J. Middleton, (I) Tacca chantrieri André, (J) Biancaea sappan (L.) Tod., (K) Carissa spinarum L., (L) Dracaena cochinchinensis (Lour.) S.C. Chen, (M) Euphorbia antiquorum L., (N) Mimusops elengi L. (heartwood), (O) Myristica fragrans Houtt., (P) Tarenna hoaensis Pit., (Q) Enhalus acoroides (L.f.) Royle, (R) Gynura pseudochina (L.) DC., (S) Kaempferia galanga L., (T) Ligusticum sinense Oliv., (U) Picrorhiza kurroa Royle ex Benth., (V) Angelica dahurica (Hoffm.) Benth. & Hook.f. ex Franch. & Sav., (W) Artemisia annua L., (X) Bouea macrophylla Griff., (Y) Citrus x aurantifolia (Christm.) Swingle, (Z) Dischidia major (Vahl) Merr., (AA) Glycyrrhiza glabra L., (AB) Heliciopsis terminalis (Kurz) Sleumer and (AC) Sophora exigua Craib.

(A)

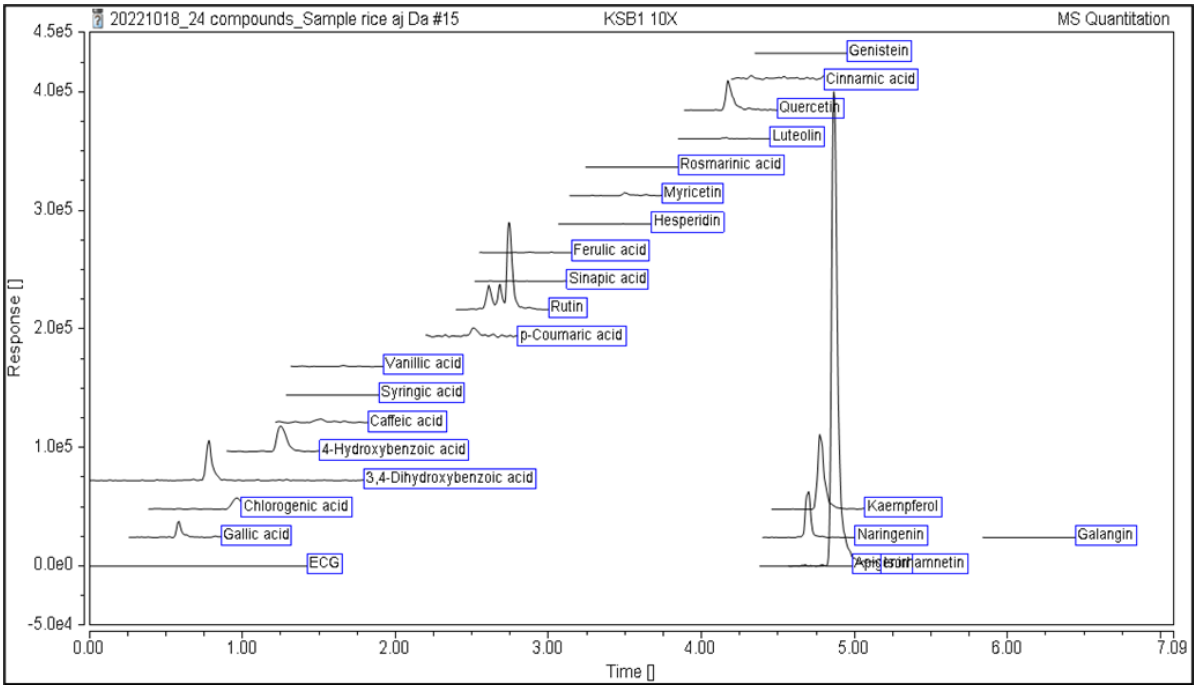

Supplementary

(B)

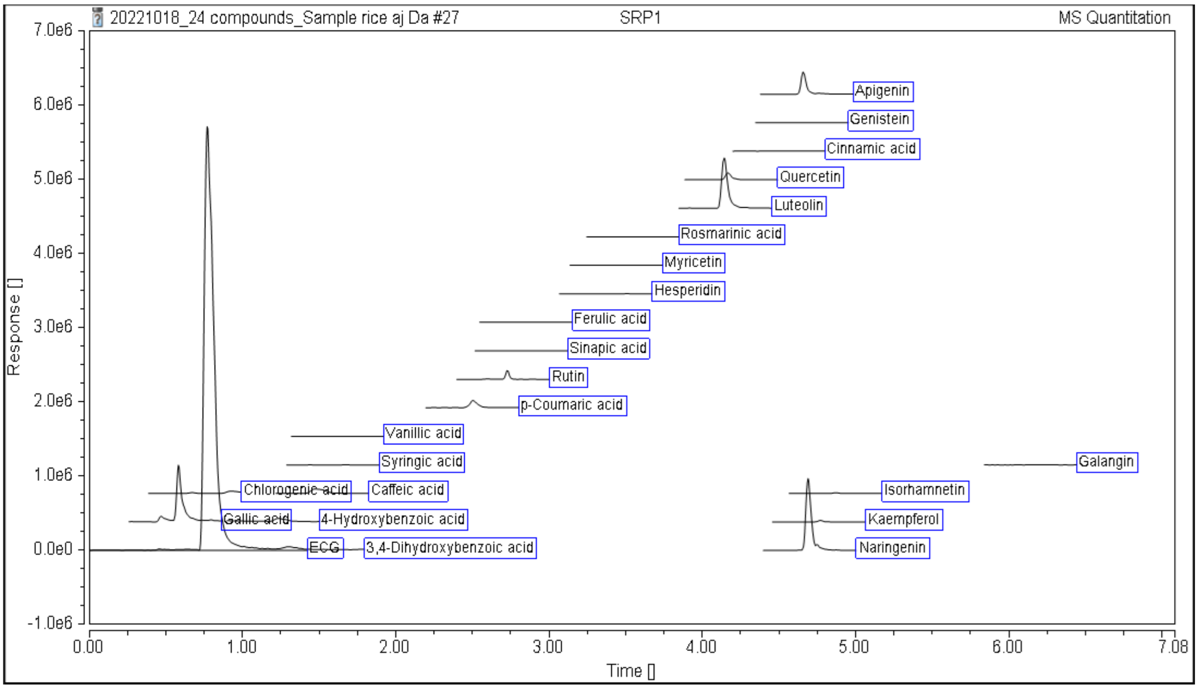

(C)

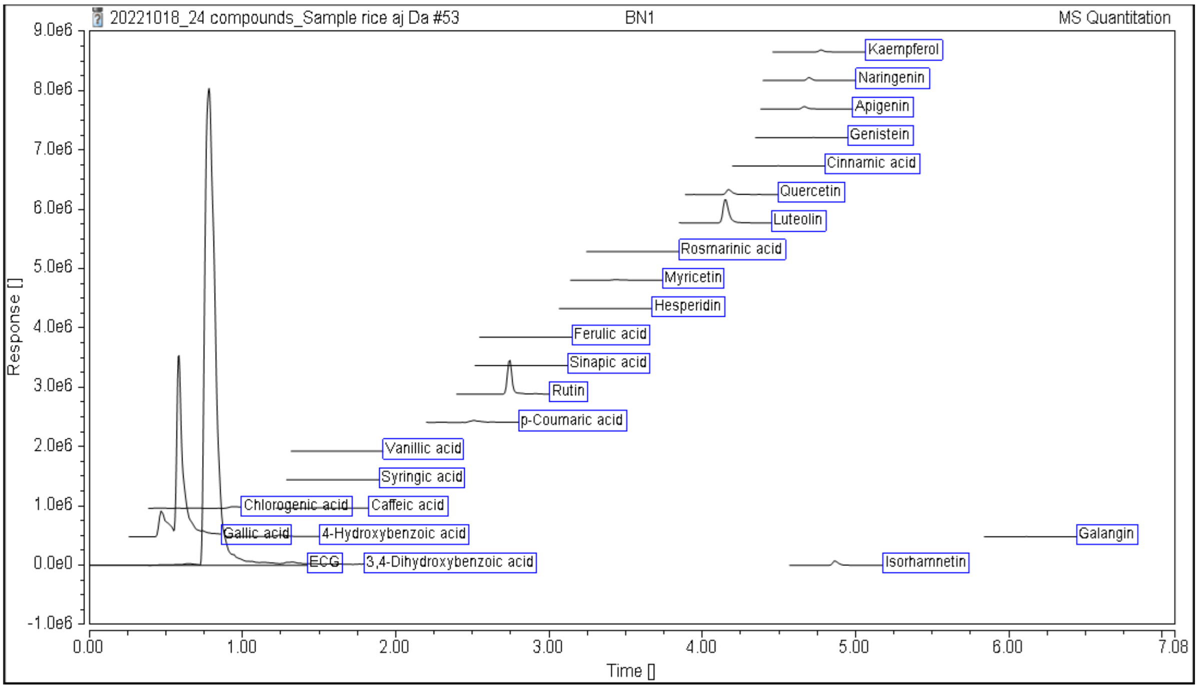

Supplementary

(D)

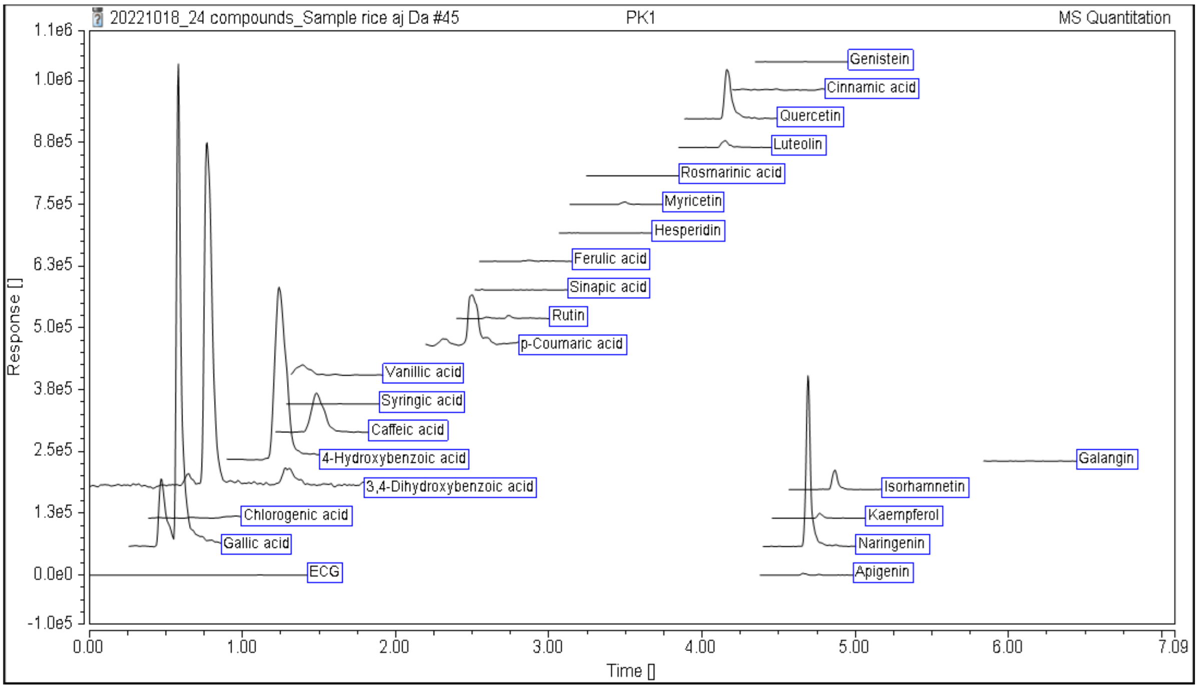

(E)

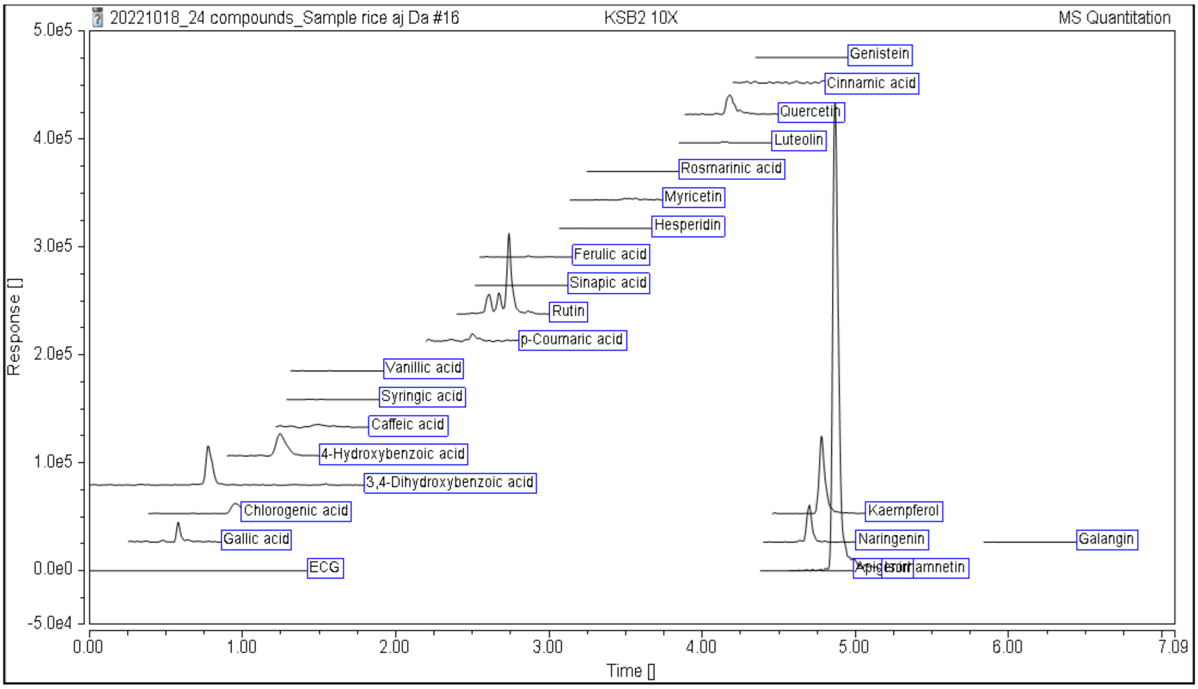

Supplementary

(F)

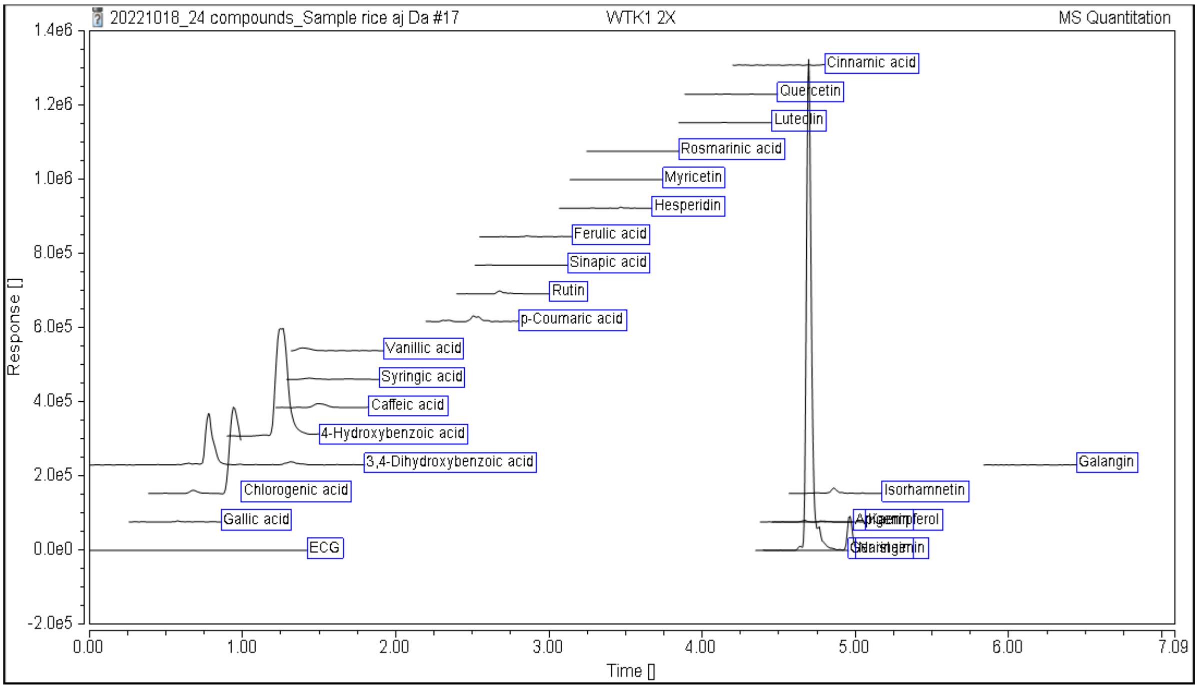

(G)

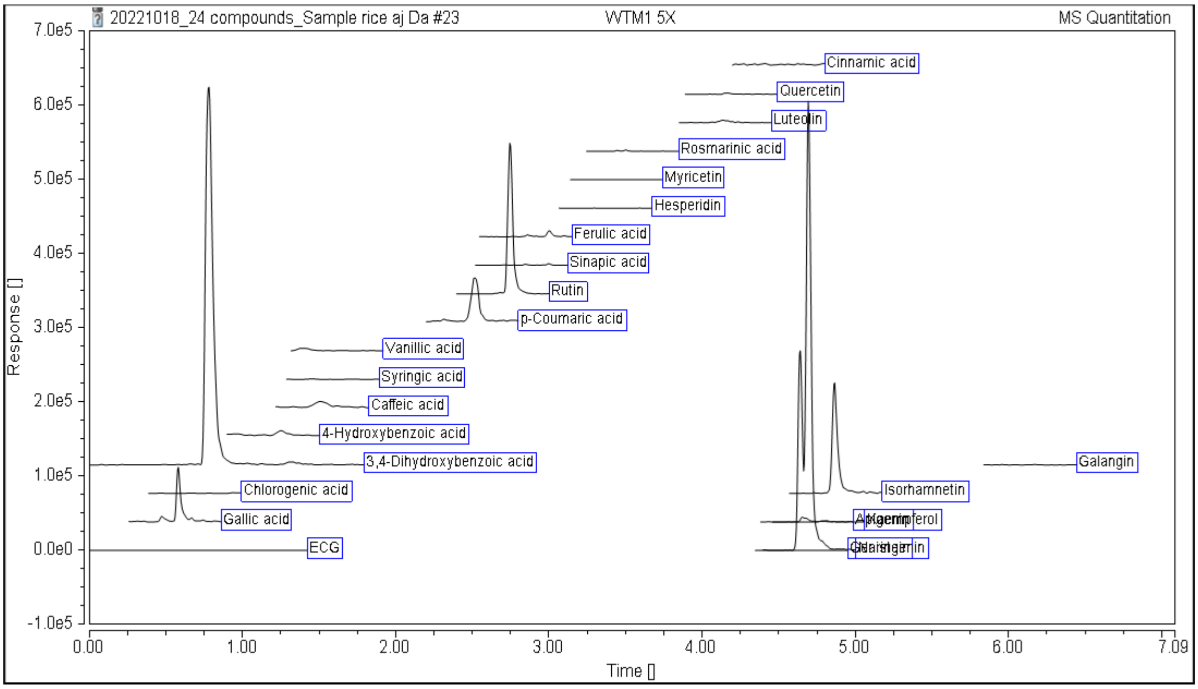

Supplementary

(H)

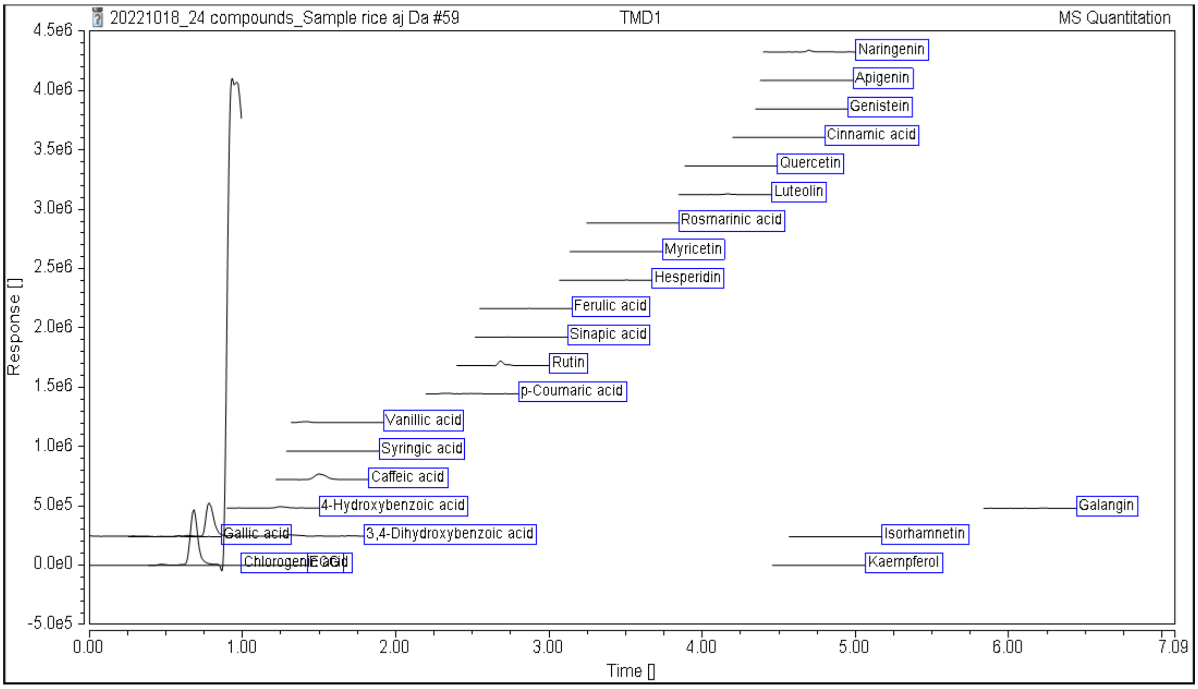

(I)

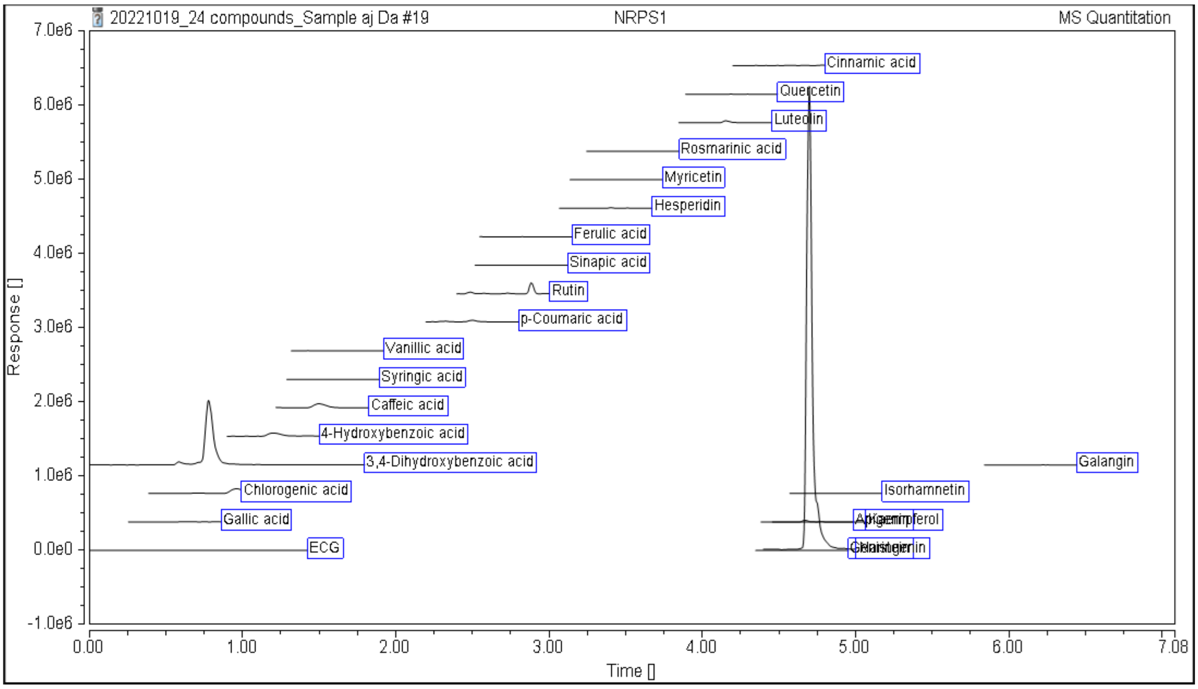

Supplementary

(J)

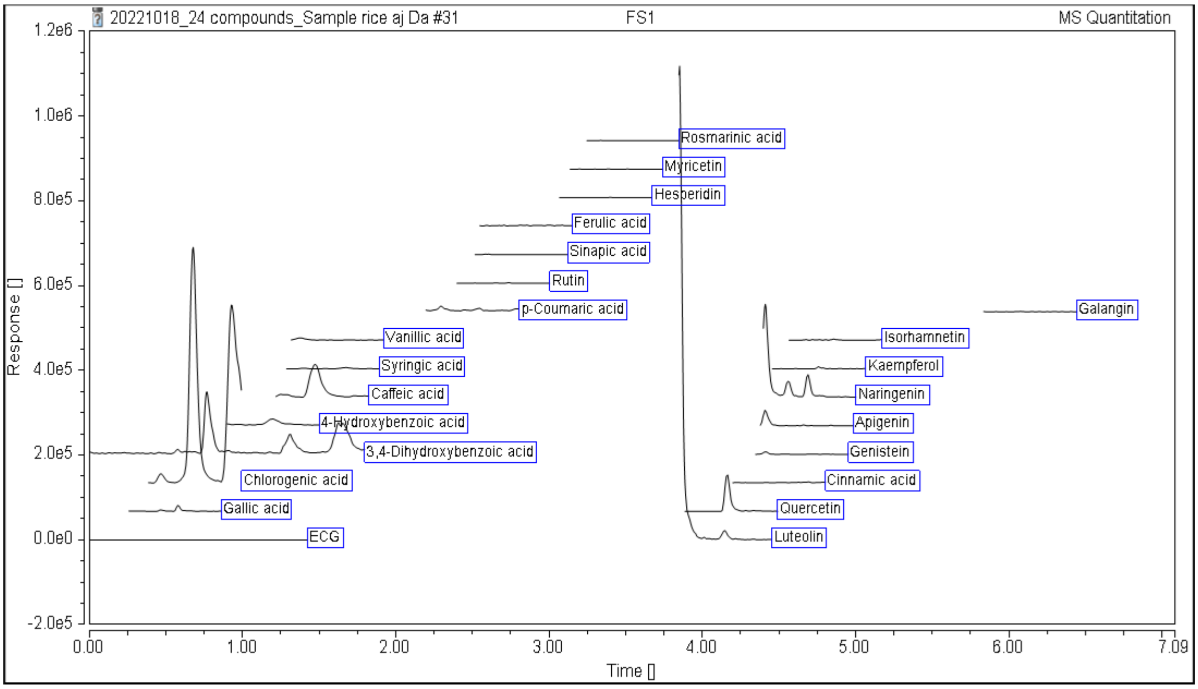

(K)

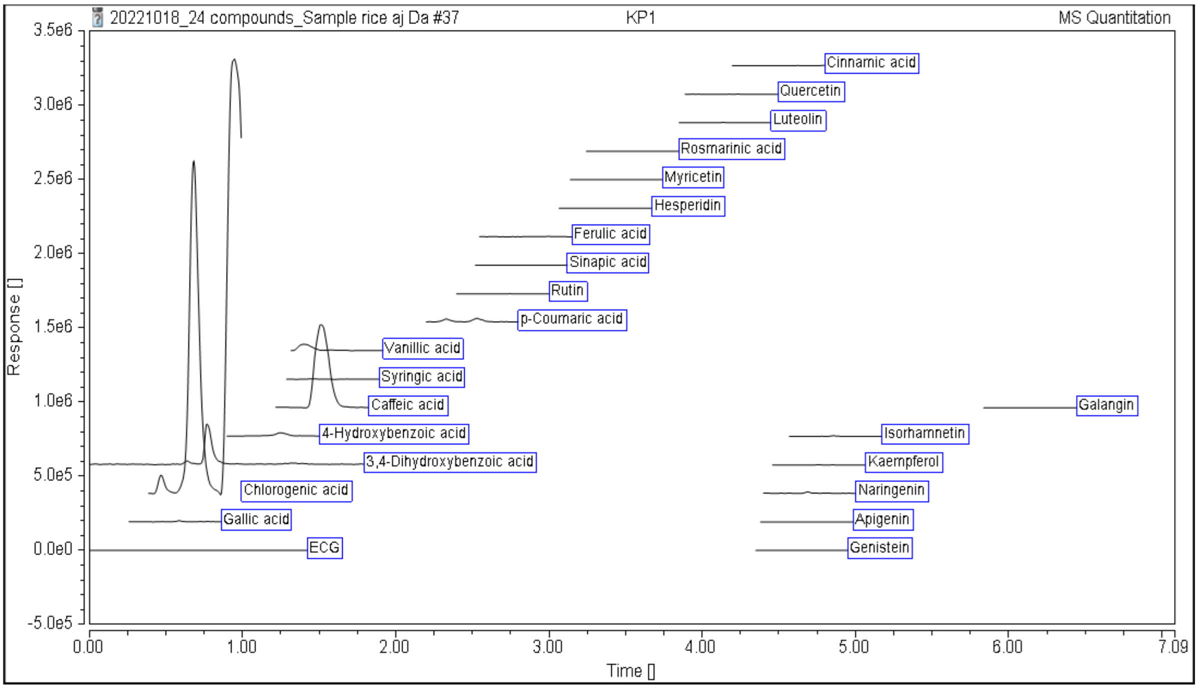

Supplementary

(L)

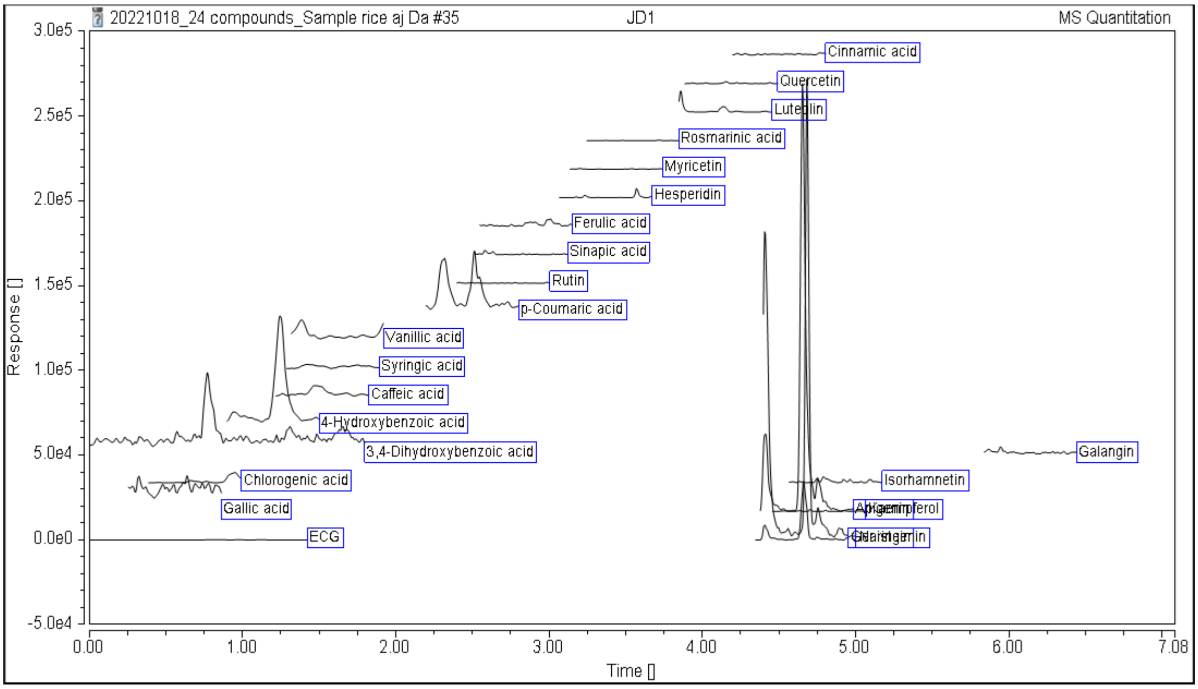

(M)

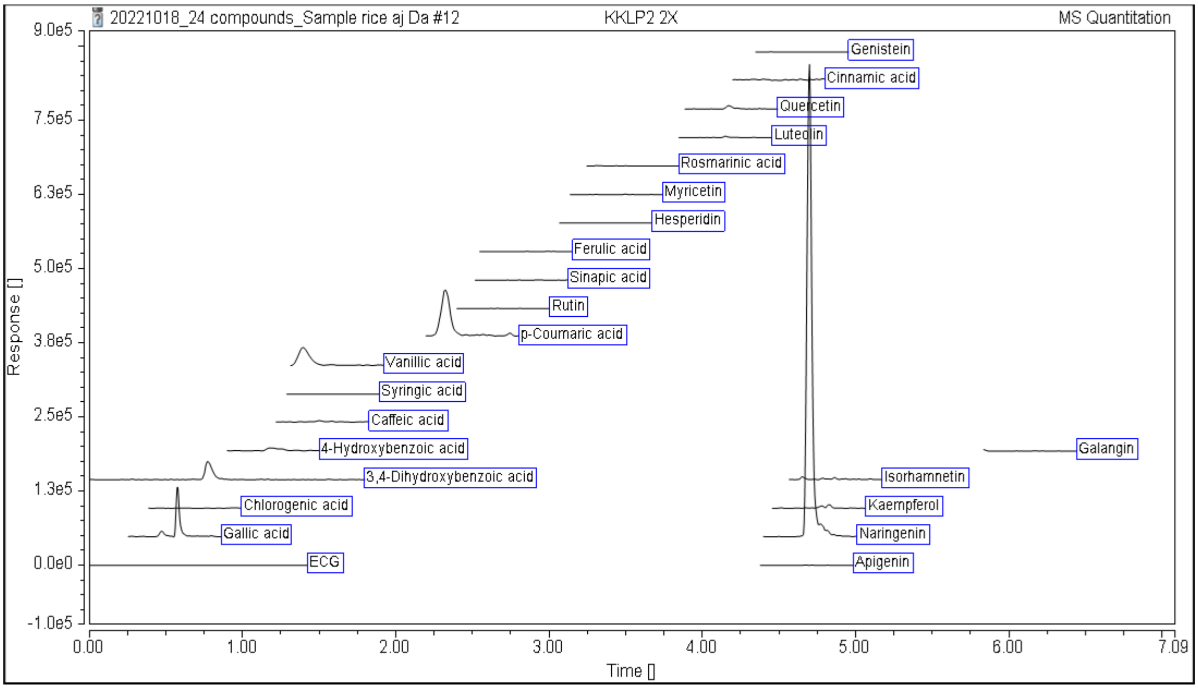

Supplementary

(N)

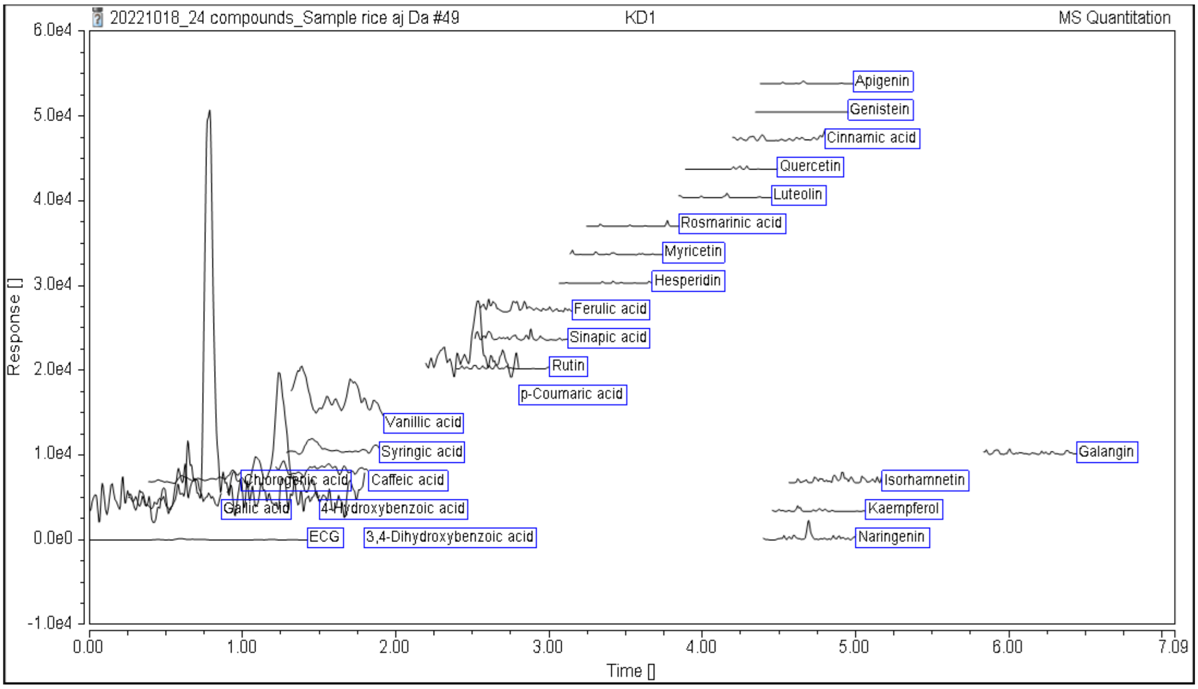

(O)

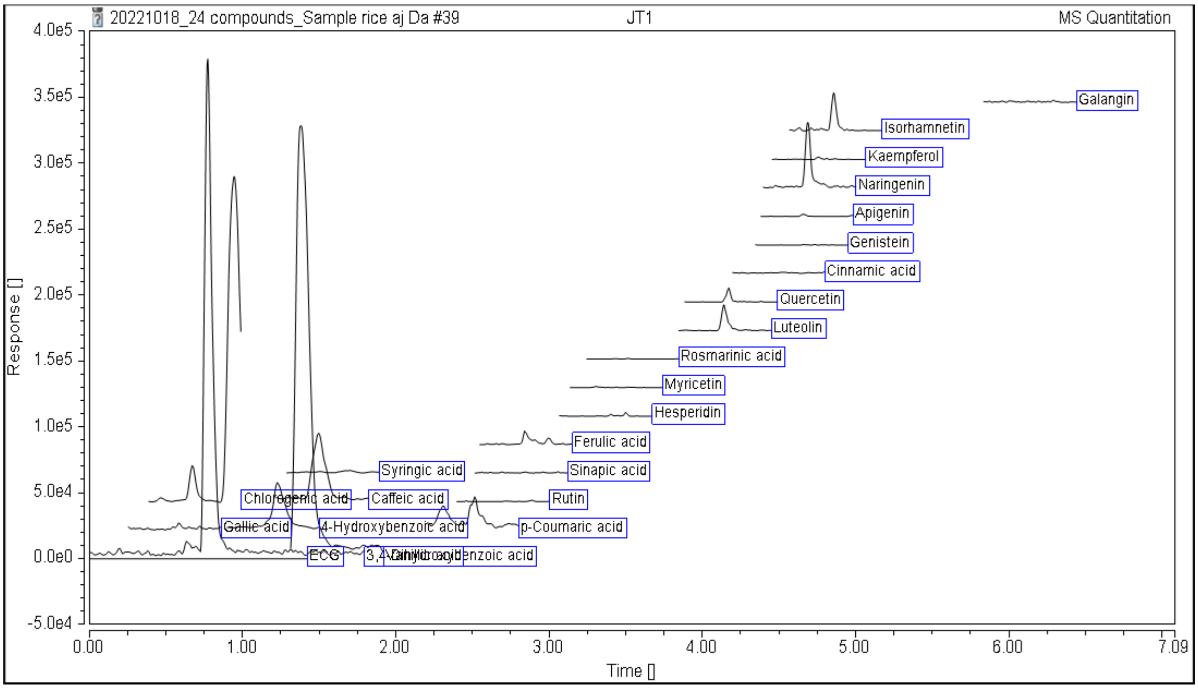

Supplementary

(P)

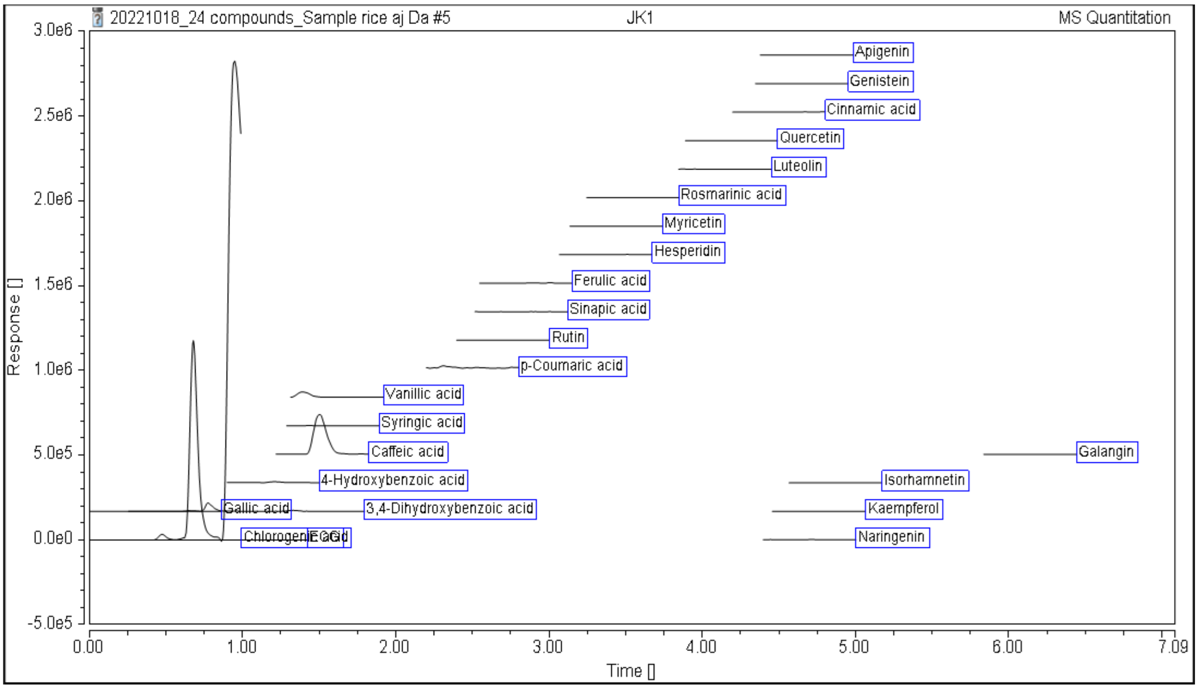

(Q)

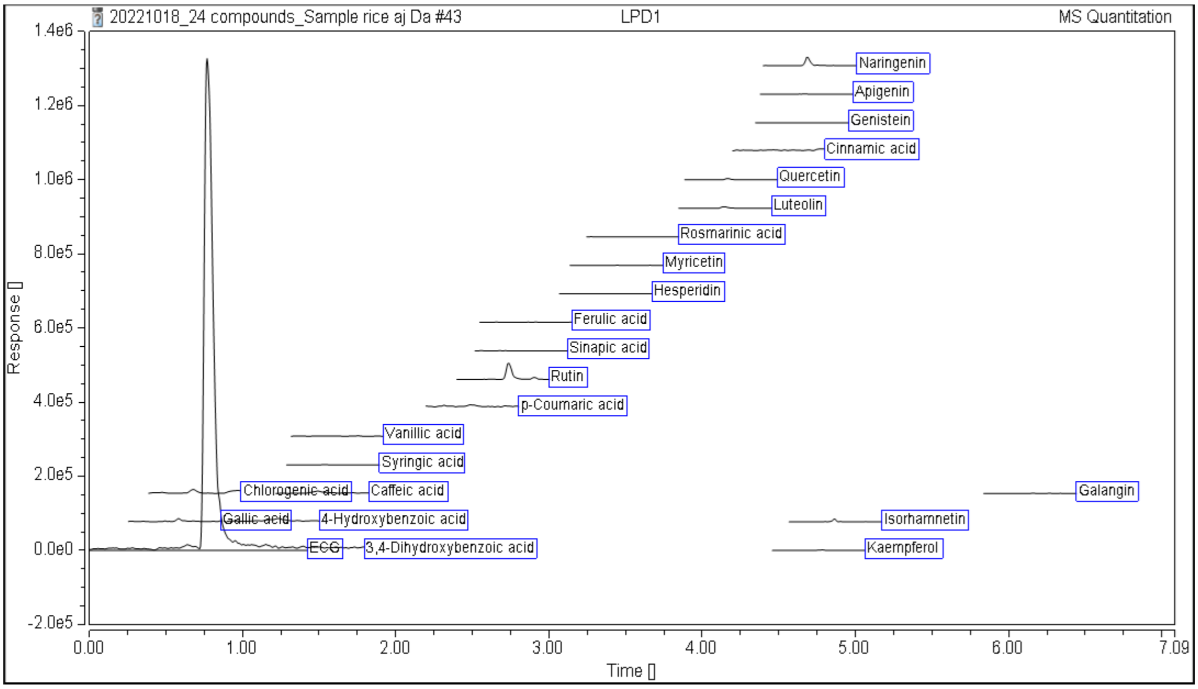

Supplementary

(R)

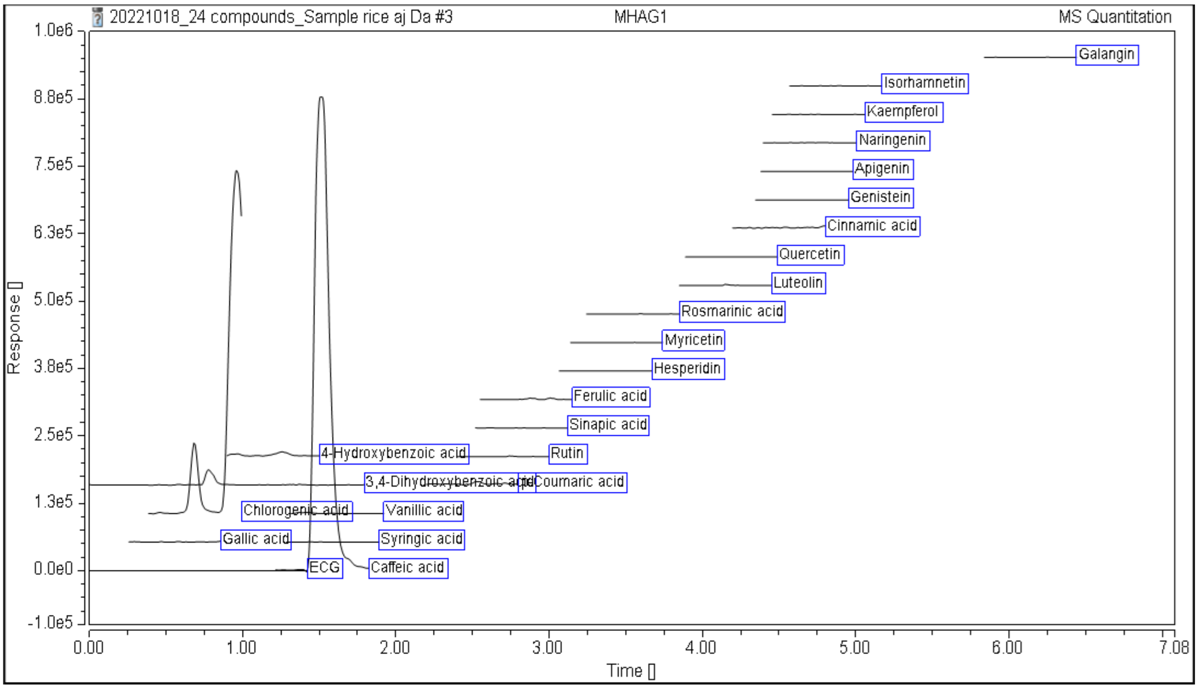

(S)

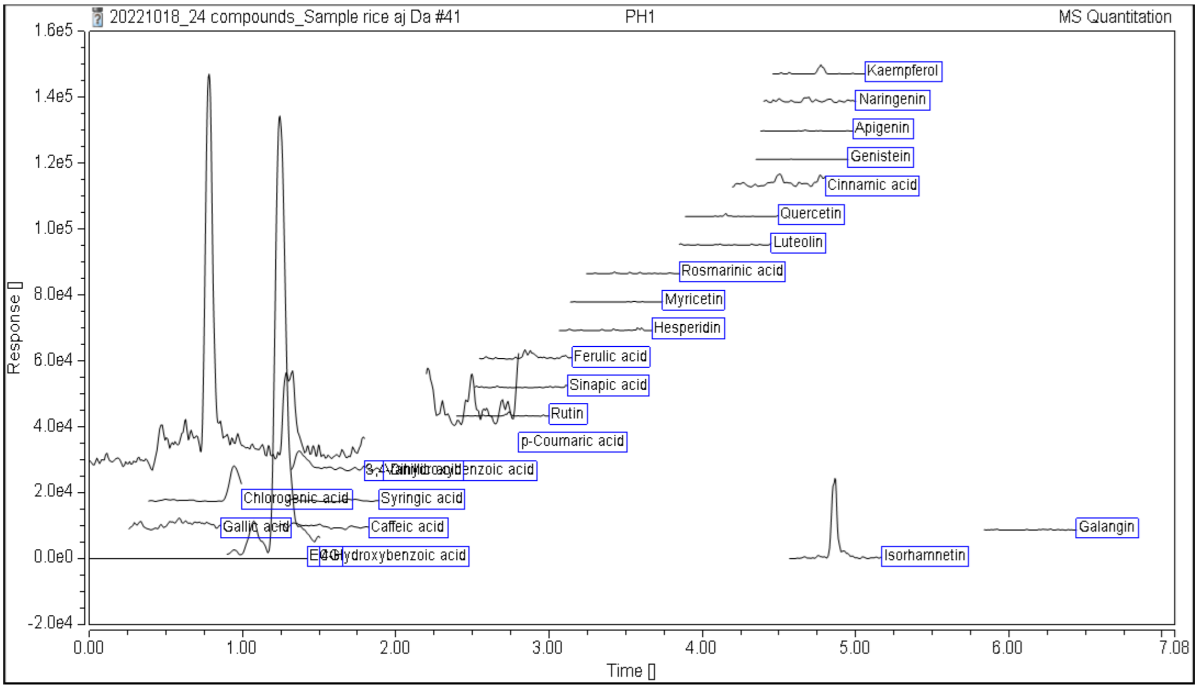

Supplementary

(T)

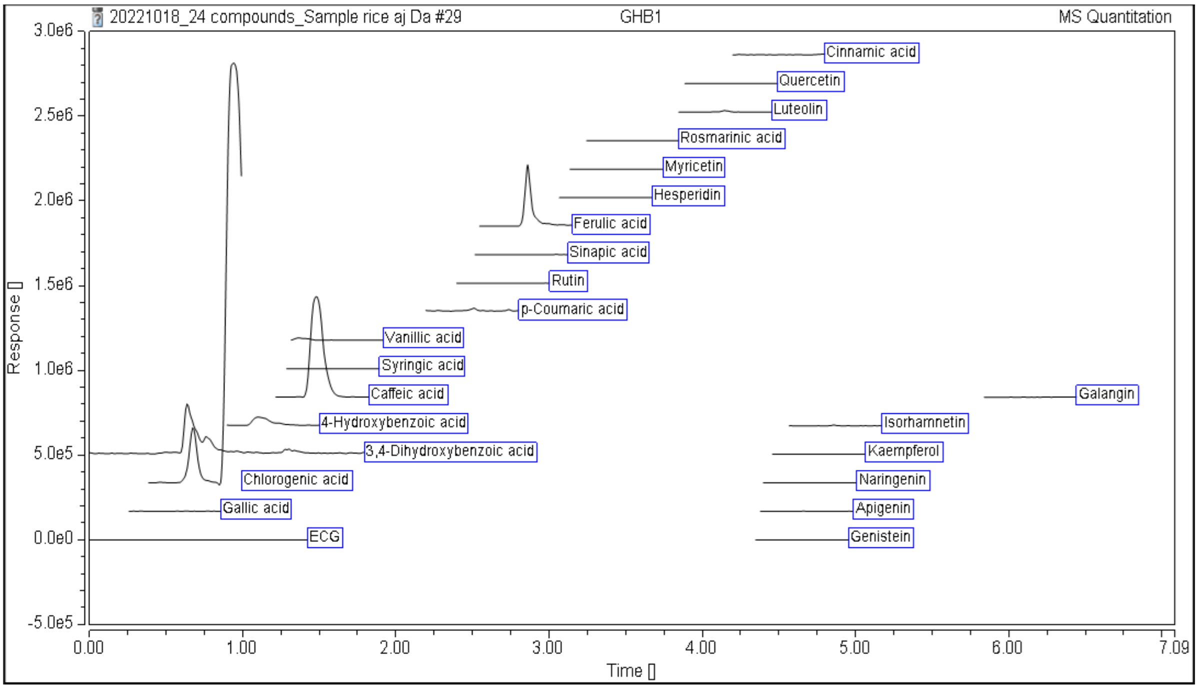

(U)

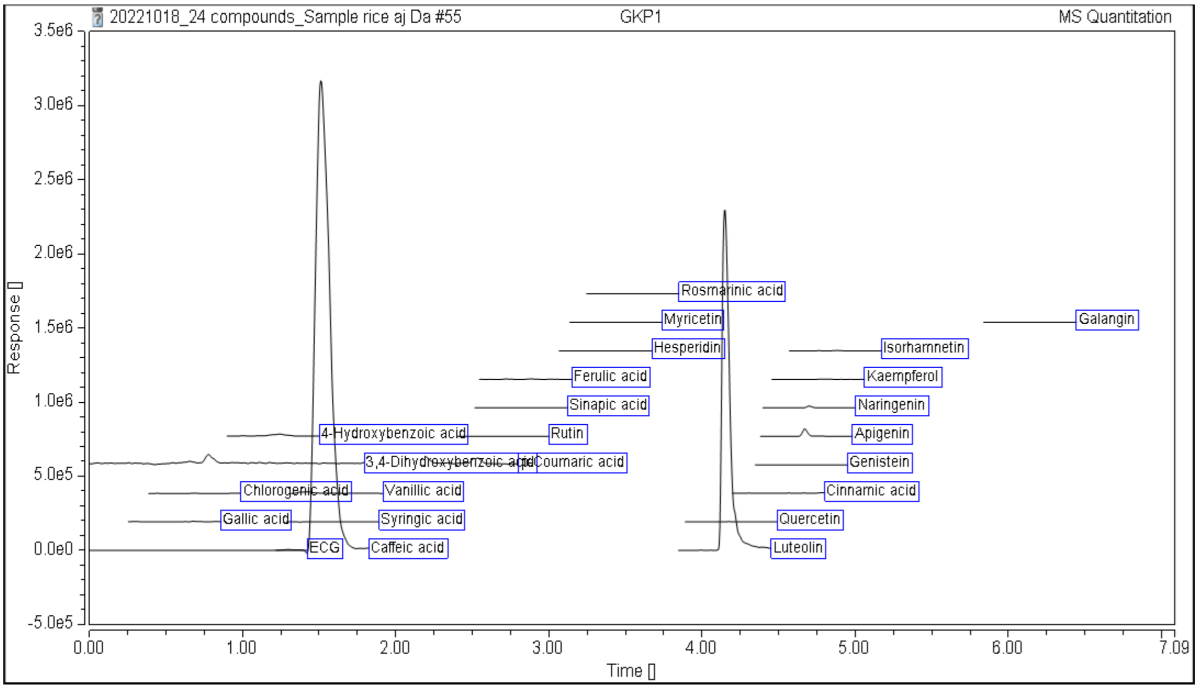

Supplementary

(V)

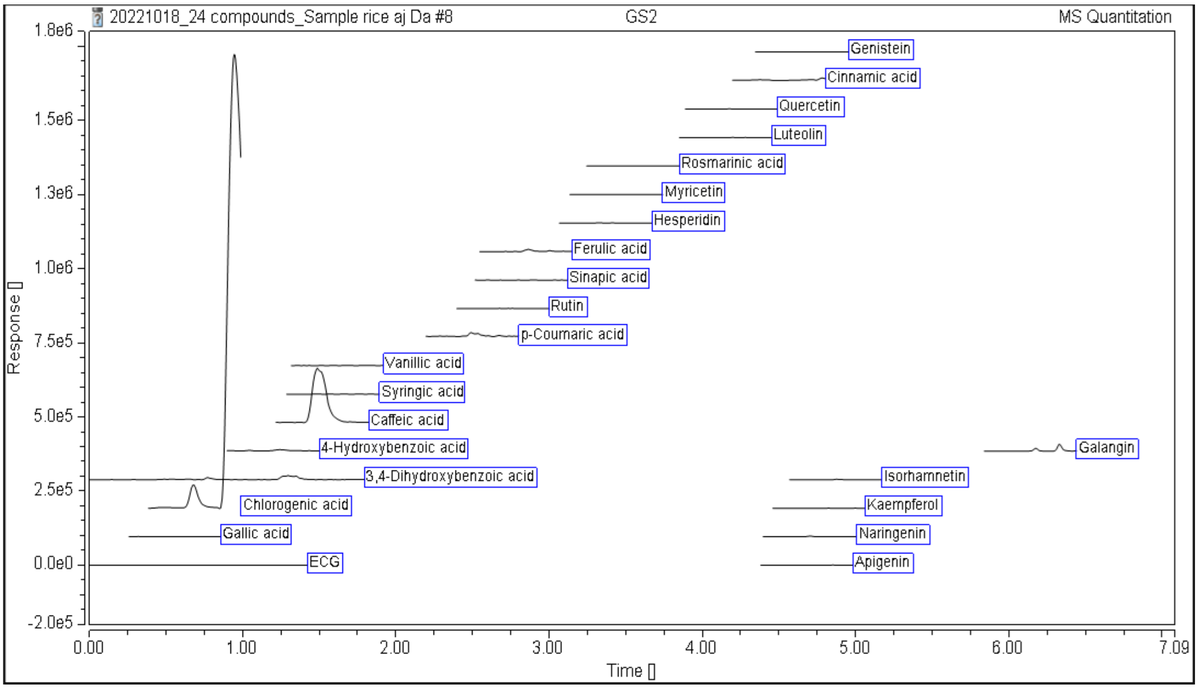

(W)

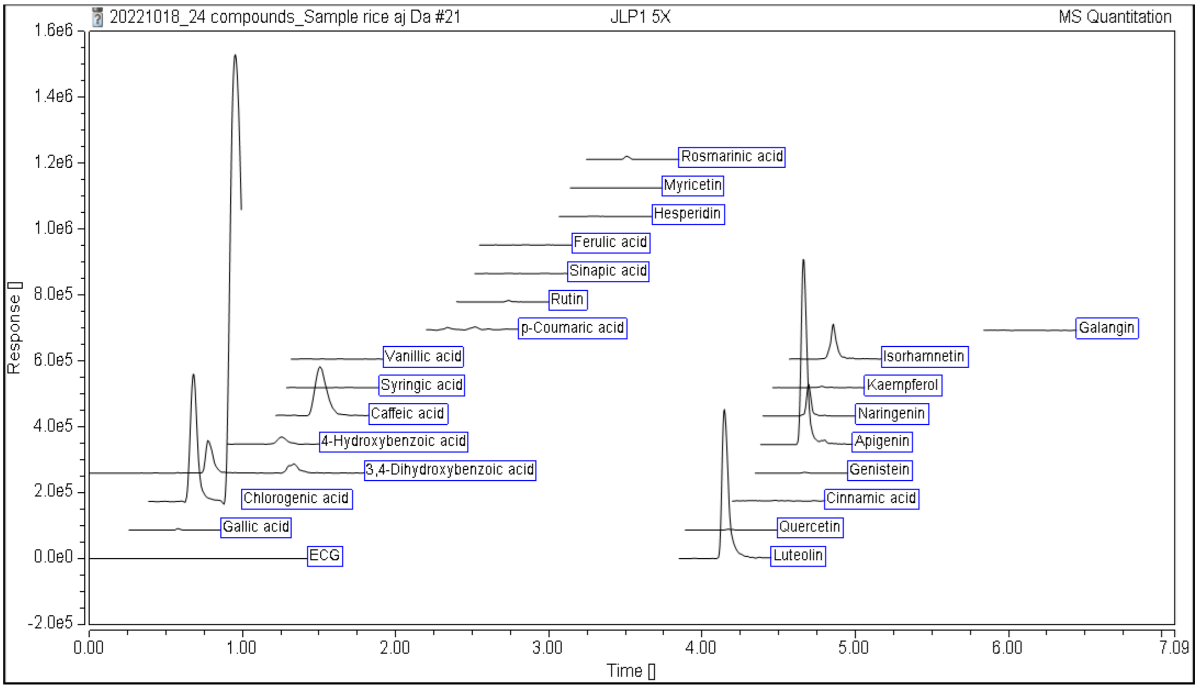

Supplementary

(X)

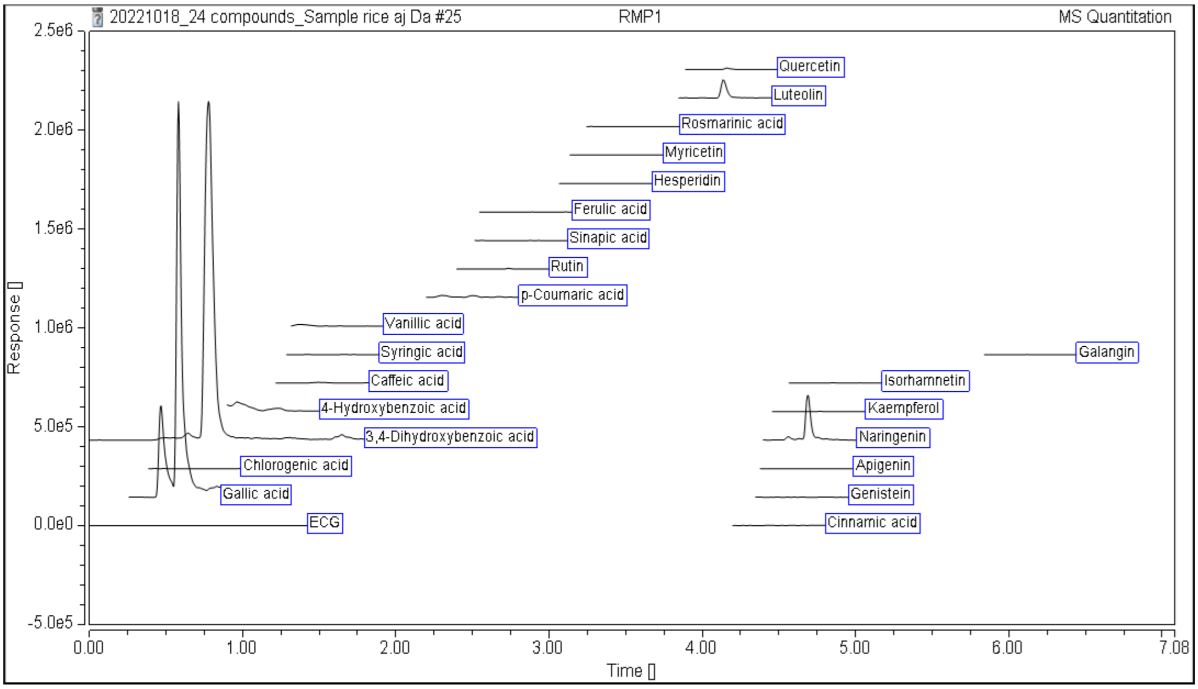

(Y)

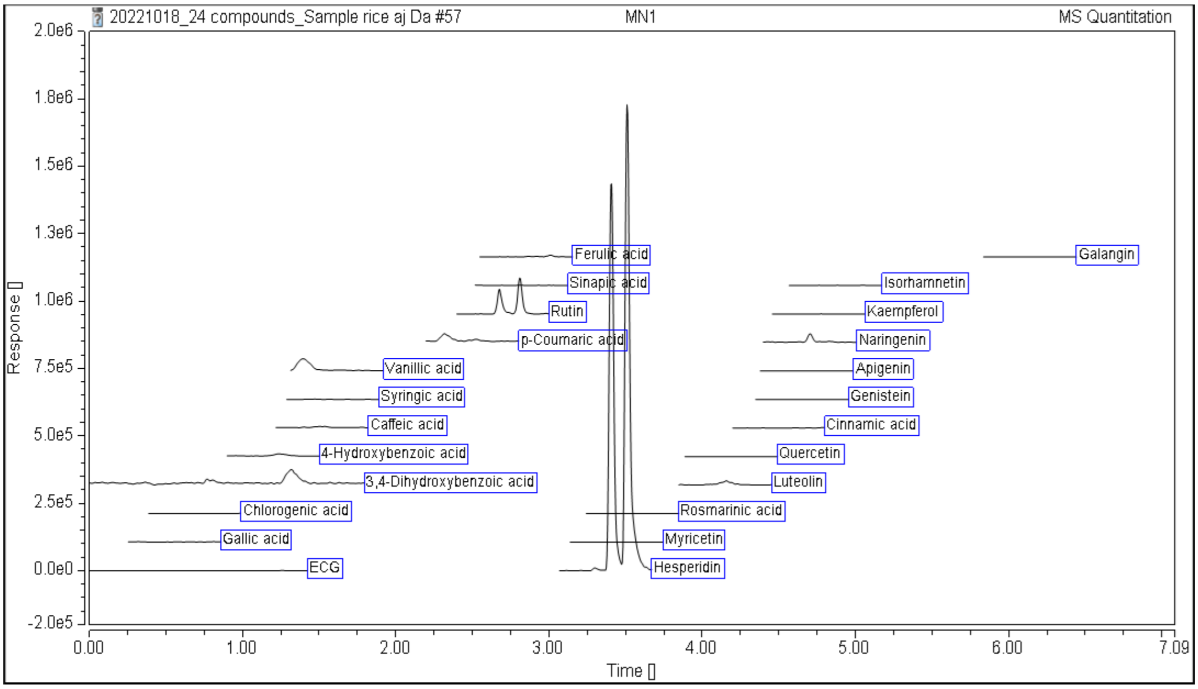

Supplementary

(Z)

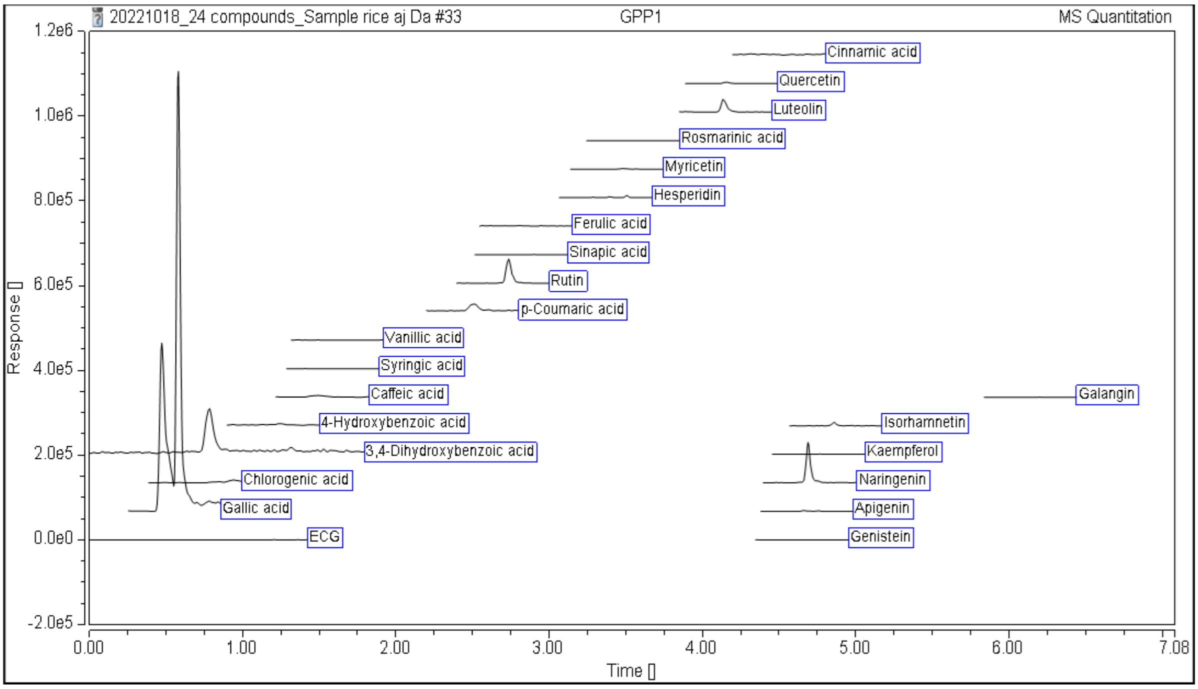

(AA)

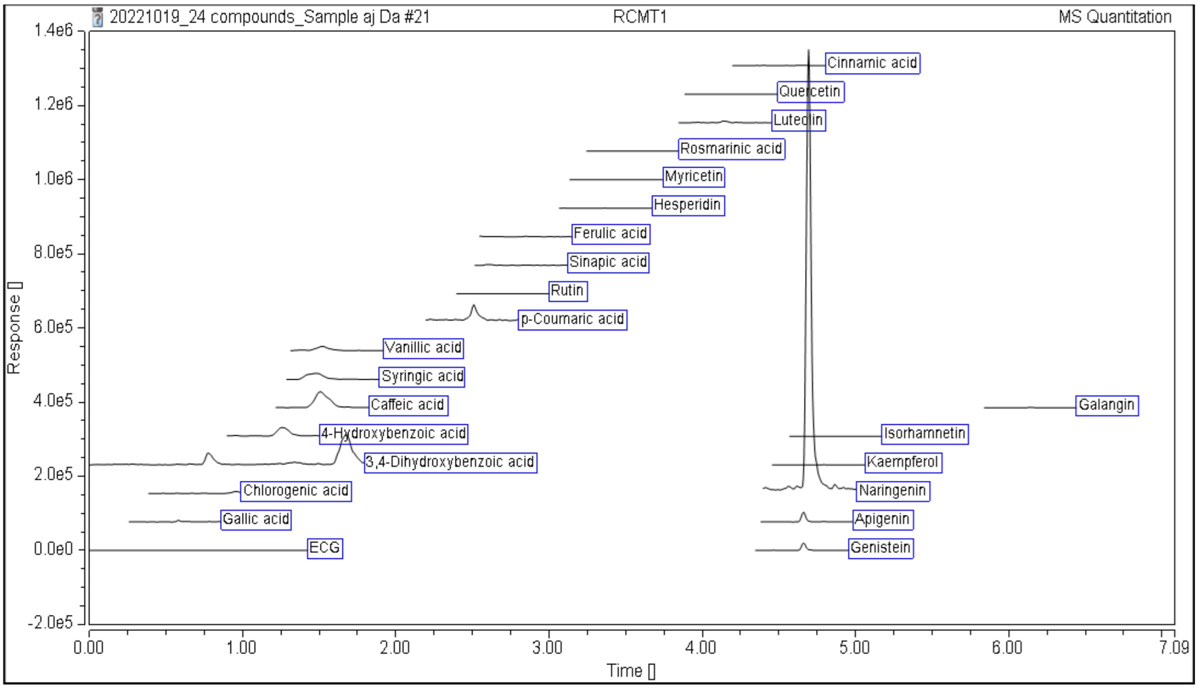

Supplementary

(AB)

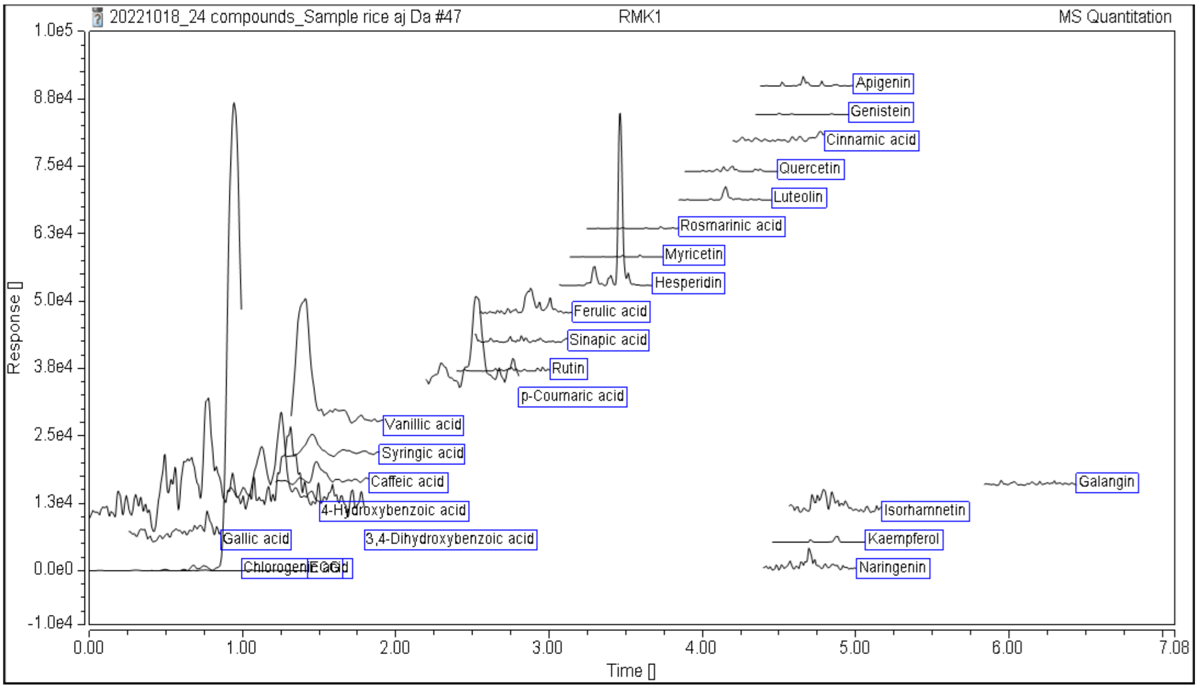

(AC)

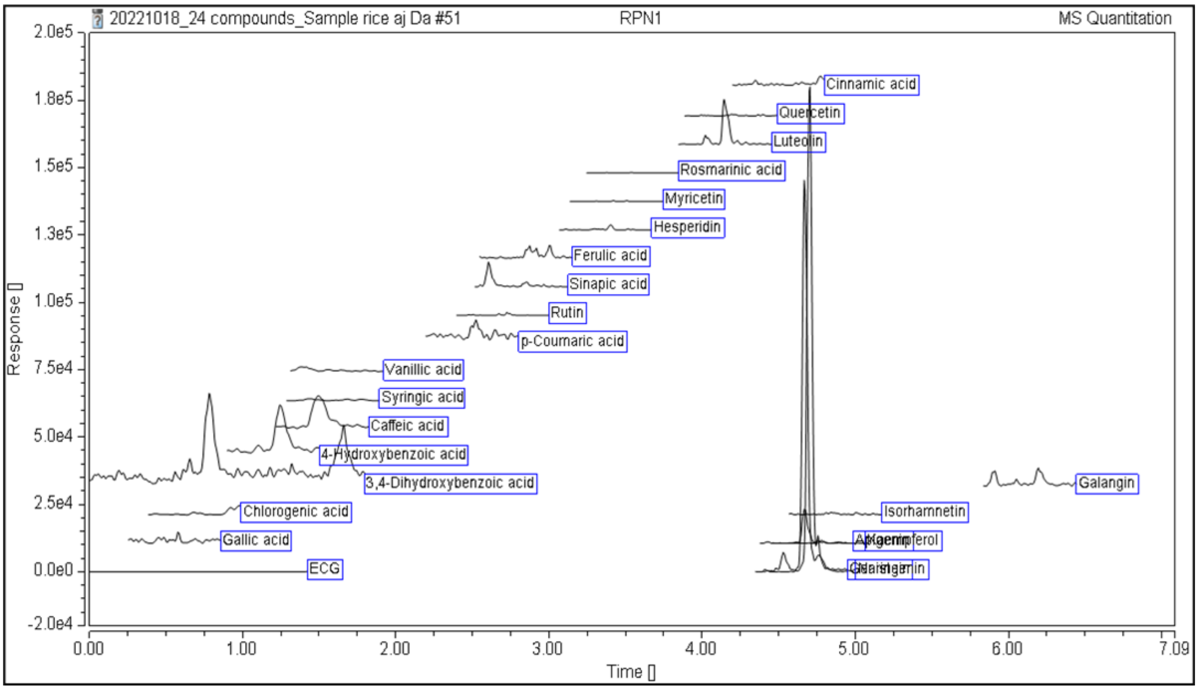

Supplement: Supplementary file 1 — Supplementary Information. [file 41598_2023_49074_MOESM1_ESM.pdf]
